# Jim Hyde and the ENDOR Connection: A Personal Account

**DOI:** 10.1007/s00723-017-0959-9

**Published:** 2017-10-23

**Authors:** Klaus Möbius, Wolfgang Lubitz, Anton Savitsky

**Affiliations:** 1 Department of Physics, Free University Berlin, Arnimallee 14, 14195 Berlin, Germany; 2 Max-Planck-Institute for Chemical Energy Conversion, 45470 Mülheim an der Ruhr, Germany

## Abstract

In this minireview, we report on our year-long EPR work, such as electron–nuclear double resonance (ENDOR), pulse electron double resonance (PELDOR) and ELDOR-detected NMR (EDNMR) at X-band and W-band microwave frequencies and magnetic fields. This report is dedicated to James S. Hyde and honors his pioneering contributions to the measurement of spin interactions in large (bio)molecules. From these interactions, detailed information is revealed on structure and dynamics of macromolecules embedded in liquid-solution or solid-state environments. New developments in pulsed microwave and sweepable cryomagnet technology as well as ultra-fast electronics for signal data handling and processing have pushed the limits of EPR spectroscopy and its multi-frequency extensions to new horizons concerning sensitivity of detection, selectivity of molecular interactions and time resolution. Among the most important advances is the upgrading of EPR to high magnetic fields, very much in analogy to what happened in NMR. The ongoing progress in EPR spectroscopy is exemplified by reviewing various multi-frequency electron–nuclear double-resonance experiments on organic radicals, light-generated donor–acceptor radical pairs in photosynthesis, and site-specifically nitroxide spin-labeled bacteriorhodopsin, the light-driven proton pump, as well as EDNMR and ENDOR on nitroxides. Signal and resolution enhancements are particularly spectacular for ENDOR, EDNMR and PELDOR on frozen-solution samples at high Zeeman fields. They provide orientation selection for disordered samples approaching single-crystal resolution at canonical *g*-tensor orientations—even for molecules with small *g*-anisotropies. Dramatic improvements of EPR detection sensitivity could be achieved, even for short-lived paramagnetic reaction intermediates. Thus, unique structural and dynamic information is revealed that can hardly be obtained by other analytical techniques. Micromolar concentrations of sample molecules have become sufficient to characterize stable and transient reaction intermediates of complex molecular systems—offering exciting applications for physicists, chemists, biochemists and molecular biologists.

## Introduction

When we were invited by the Guest Editors of this AMR Special Issue to contribute a paper in honor of James S. Hyde, we felt excited to be among those who show their respect to such an outstanding EPR jubilarian. We know Jim Hyde since a long time, one of us (K.M.) even for more than 50 years, experiencing him always as one of the leading magnetic-resonance scientists, a stimulating colleague—and noble contestant in advanced ENDOR spectroscopy.

Jim Hyde is the founder of the National Biomedical EPR Center at the Medical College of Wisconsin in Milwaukee, a US Research Resource supported by NIH. He served as Director of the EPR Center from 1980 until his retirement in 2016. The Center grant has been continuously funded since 1976. Forty years of continuous public funding—that’s a success story in itself.

Jim Hyde’s research interests in EPR partly overlapped with our own research interests: to develop advanced EPR instrumentation with exciting applications to extend the ways in which EPR spectroscopy can be used for new categories of biomedical problems that are not yet accessible by standard EPR or other conventional techniques. Key techniques in this endeavor are double-resonance methods in a variety of microwave-frequency, magnetic-field combinations, such as ENDOR and ELDOR at X-band and W-band. Interesting spin physics and spin chemistry of biocomplexes could thus be studied, such as proteins and membranes embedded in their natural or artificial matrices, to elucidate their structure, dynamics and function relationships. By using site-directed spin labeling, SDSL, with tailor-made nitroxide spin labels, the applicability of EPR methods in biological sciences has been strongly widened. The SDSL methodology holds great promise in many areas of the life sciences, it was pioneered by Wayne Hubbell and co-workers at UCLA, and Jim Hyde and his colleagues at the Medical College of Wisconsin are among the frequent users of this methodology.

For this *Festschrift* we decided to contribute a mini-review of ENDOR-related papers from many years reporting on exciting studies that more or less remained related to Jim Hyde’s own interests in science, for example liquid-solution and frozen-solution ENDOR, high-field EPR, ENDOR, EDNMR and PELDOR on orientation selected powder-type paramagnetic and nitroxide spin-labeled biosystems.

## The Early Years of the ENDOR Connection

The first encounter with Jim Hyde was in 1965 when one of us (K.M.) attended the international EUCHEM Conference on “Chemical Aspects of Electron Spin Resonance” at the Royal Agricultural College in Cirencester (England). The attendees stayed on site, with a cup of early-morning tea served in the dormitory’s bedrooms. Excellent. It was the first-ever EUCHEM Conference, opening a long series of subsequent EUCHEM Conferences around Europe that last until today.

**Fig. 1:**
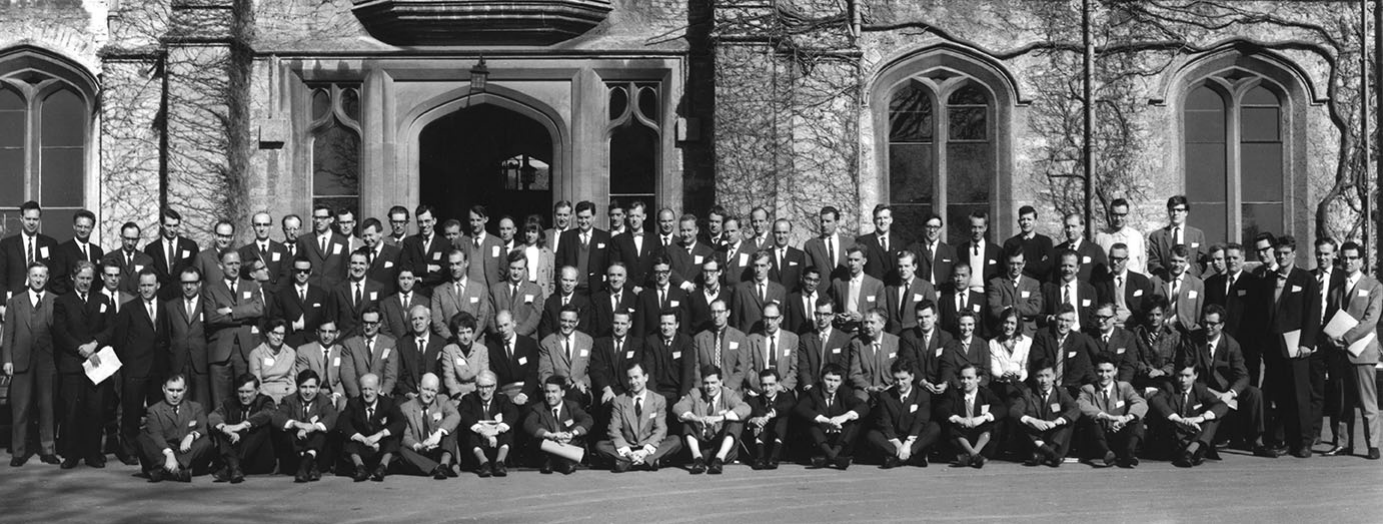
First EUCHEM Conference on “Chemical Aspects of Electron Spin Resonance” at the Royal Agricultural College in Cirencester (England), March 28–April 2, 1965, organized by David H. Whiffen (National Physics Laboratory, NPL, Teddington, Middlesex). Participants [1]: from left to right, 1st row, sitting: A. Horsfield (1), J.H. Freed (3), E. Warhurst (4), B. Mile (5), A.G. Evans (6), C. Corvaja (7), B.C. Gilbert (16); 2nd row, sitting: F. Gerson (2), R.O.C. Norman (3), W.A. Waters (4), V.V. Voevodsky (6), J.S. Hyde (7), R.E. Richards (8), A. Carrington (9), D.H. Whiffen (10), G.K. Fraenkel (11), A.J. Stone (12), H.C. Longuet-Higgins (13), E. Wasserman (14), G. Giacometti (20); 3rd row, standing: D.D. Eley (2), K. Möbius (largely hidden, 3), J.H. van der Waals (6), P.B. Ayscough (11), G.R. Luckhurst (16), N.M. Atherton (17), A. Hinchcliffe (20), A.H. Reddoch (22), J. Kommandeur (23), J.D.W. van Voorst (24), G. Schoffa (27), H. Fischer (largely hidden, 28), R.J. Cook (30), E. de Boer (31); 4th row, standing: M. Plato (2), J.E. Bennett (4), R.W. Fessenden (largely hidden, 9), C. Lagercrantz (largely hidden, 11), J.R. Bolton (12), J.C. Evans (18), W. Müller-Warmuth (24), R.G. Brandon (31), H. Bär (32), J.R. Morton (33), D.P. Santry (34)

The Cirencester symposium was in fact K.M.’s first international conference (as it was Jim Hyde’s). It started only 3 weeks after his wife Uta gave birth to their first child, and it was not easy for the young parents to convince each other that K.M. would take a leave of absence just for attending an EPR meeting in Cirencester. Eventually, however, K.M. felt always obliged to David Whiffen (1922–2002) for having catalyzed, via the Cirencester conference, the opportunity to personally meet with so many fascinating EPR scientists, among them first and foremost Jim Hyde, Jack Freed, George Fraenkel, Anthony Stone, Fabian Gerson, Giovanni Giacometti. Reading now the names in the figure caption is somehow like reading *Who’s Who* in the foundation years of modern EPR [2]. Later, David Whiffen became a source of inspiration for us for our electron–nuclear–nuclear triple-resonance experiments [3] (Fig. 1).

In the program of the meeting, K.M. gave a (short) lecture on his precision measurements of *g*-factors of aromatic charged and neutral radicals in fluid solution with a relative accuracy of ± 2 × 10^−6^. These challenging measurements were part of his Ph.D. project at FU Berlin, which he had just submitted for publication (March 23, 1965) [4], aimed at testing the recent theoretical *g* value predictions of A.J. Stone from Cambridge University [5]. Anthony Stone was in the audience, and he seemed to be pleased that the high-precision *g*-factor data indeed verify his theoretical prediction concerning their linear dependence on the radicals’ Hückel orbital energies of their lowest half-occupied π-orbital. As was, of course, K.M. after his lecture. To his surprise he then learned from George Fraenkel in the audience that analogous precision *g*-factor measurements had just been carried out independently in his group at Columbia University by Bernice Segal and Michael Kaplan—with essentially similar results as ours at FU Berlin. Naturally, we exchanged our *g* value results prior to publication [6], their paper was submitted August 9, 1965.

**Fig. 2:**
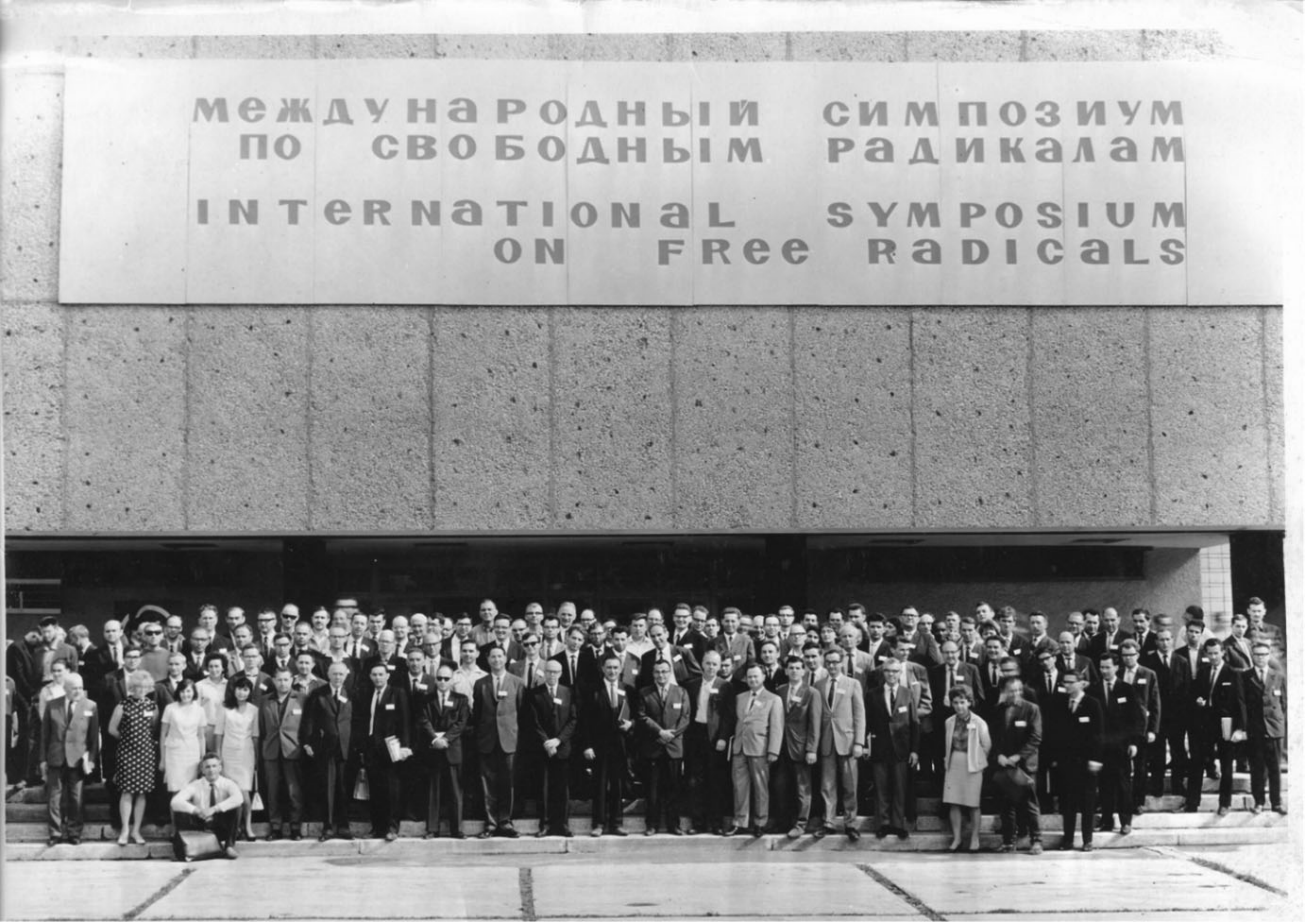
The eighth International Symposium on Free Radicals, July 26–31, 1967, Novosibirsk (Akademgorodok), USSR

The mid-1960s had been remarkable years in many aspects, also in politics: in 1964, the USSR leader Nikita Khrushchev had been driven out of power of the Soviet Union. His communist party colleagues replaced him with Leonid Brezhnev as First Secretary of the Communist Party and Alexei Kosygin as Premier. Khrushchev had led the Soviet Union during icy periods of the Cold War. But towards the early 1960s there was some softening in the frozen East–West relationship that was encouraged by Khrushchev and, subsequently, continued by Brezhnev. As one of the results of the thawing relations, Soviet scientists from ideologically “innocent” areas like molecular spectroscopy were permitted by their authorities to attend scientific meetings in Western countries. Taking this chance, Academician Vladislav V. Voevodsky (1917–1967), one of the leading figures in Soviet physical chemistry research, was invited by David Whiffen to participate in the Cirencester conference. Six months later, Voevodsky became also a prominent participant of the seventh International Symposium on Free Radicals in Padova, Italy, September 5–10, 1965, which was organized by Giovanni Giacometti and Giovanni Semerano. At that occasion, Voevodsky was elected by the Steering Committee to organize the follow-up 8th International Symposium on Free Radicals in Novosibirsk, USSR; it was scheduled for July 1967 (Fig. 2).

Voevodsky was an extremely important person in chemical and biological spectroscopy in the USSR, in particular for promoting the application of EPR in Soviet science institutions. EPR had been badly neglected since Zavoisky’s discovery of EPR in Kazan in 1944. What a sad example of ideology-driven self-mutilation!

In 2017, the international magnetic-resonance community remembered the 110th anniversary of Evgeni K. Zavoisky and 100th anniversary of Vladislav V. Voevodsky. And it is only 2 years ago that we remembered the 80th anniversary of Yakov S. Lebedev, the pioneer of high-field EPR spectroscopy. A few years ago the EPR community celebrated the 80th birthdays of Yuri D. Tsvetkov (born 1933) and Yuri N. Molin (born 1934) who shaped, after Voevodsky’s much too early death, the research areas of the Institute of Kinetics and Combustion in Novosibirsk. And 1 year ago, we celebrated the 80th birthday of Kev M. Salikhov, the theoretician of spin dynamics and spin chemistry—and the pioneer of electron–electron dipolar spectroscopy PELDOR. In 1989, Kev Salikhov had been elected head of the E.K. Zavoisky Physical-Technical Institute in Kazan, an eminent research institution of the Russian Academy of Sciences.

Nowadays, the EPR scientists from Kazan, Novosibirsk, Moscow or elsewhere in the former Soviet Union are natural part of our common magnetic-resonance world heritage, with many international scientific journals reporting on new and historic achievements, across old borders and lasting ideologies. But honestly, it took quite a while until this EPR-related glasnost could happen. Hence, it is about time, also for this *Festschrift,* to briefly recall the “thorny path” [7] to the advent of biological and chemical EPR spectroscopy in the Soviet Union. Even more so since Jim Hyde belongs to the group of scientist who had an important share in the efforts to smooth this thorny path.

Among the few prominent scientists from academia who repeatedly asked for full-scale support of EPR and NMR research in the USSR, four persons have to be specifically praised: structural chemist Academician Jakov K. Syrkin (1894–1974), Nobel laureate Nikolai N. Semenov (1896–1986), physical chemist Academician Vladislav V. Voevodsky (1917–1967), and biophysicist Lev A. Blumenfeld (1921–2002). They played key roles in introducing the magnetic-resonance methods into biological and chemical sciences in the USSR [7].

In 1955, a committee headed by V.V. Voevodsky was appointed by the Academy of Sciences of the USSR to extend the efforts to develop magnetic resonance (both EPR and NMR) at the famed Institute of Chemical Physics in Moscow. Because of Western embargo restrictions, these efforts included the in-country development of equipment for microwave generation and detection, magnets and power supplies. Emphasis was put also on stepping up the theoretical efforts in the USSR for mastering the knowledge of sensitivity limits achievable for magnetic-resonance instrumentation. A year later, in 1956, Voevodsky employed the highly talented engineer Anatoly G. Semenov (1924–1990) for EPR instrumentation development. Later, he indeed developed EPR spectrometers with good sensitivity performance for many applications (as long as the spin concentration was high enough).

In 1957, the Siberian Branch of the Academy of Sciences of the USSR had been established under the leadership of the mathematician Mikhail A. Lavrentyev (1900–1980). The foundation of Siberia’s “Academic Town” Akademgorodok (soon a district of Novosibirsk) remains his most widely known achievement. V.V. Voevodsky was chosen to build up a potent physical-chemistry research group. He moved from Moscow to the Institute of Kinetics and Combustion in Akademgorodok with his highly capable and motivated research associates Yu.N. Molin and Yu.D. Tsvetkov. Together with the theoretician Kev M. Salikhov (born in 1936), Yuri Tsvetkov established a new scientific school of chemical magnetic spectroscopy. They included Anatoly G. Semenov, the excellent instrument builder, in the group who thereafter led the technical development and production of a number of specialized EPR and NMR spectrometers for various fundamental and applied tasks that served well for many years in numerous Soviet laboratories. For this strategic goal to achieve, and this without the option to buy EPR instrumentation from Western companies like Varian Associates, Palo Alto, USA, Voevodsky had somehow managed to organize the serial production of about a hundred Siberian EPR spectrometers of the famous type “EPR-3, Sibir” (“ЭПP-3, Cибиpь”) developed by A.A. Semenov. Voevodsky was the right person to carry through this Herkulean task. He was a charismatic leader who could convince his co-workers to try unconventional methods rather than to plainly give orders.

While Yu.N. Molin and Yu.D. Tsvetkov had moved to Novosibirsk, another highly gifted disciple of V.V. Voevodsky, Yakov S. Lebedev (1935–1996), stayed in Moscow to start a dedicated research program on pioneering high-field/high-frequency EPR in physical chemistry [8, 9]. The success of his program was a great step forward in advanced EPR spectroscopy, and in fact high-field/high-frequency EPR—together with pulsed EPR—became the essential ingredients of the success story which put EPR into the position of unstoppably catching up with NMR in modern magnetic-resonance spectroscopy. Notably, for both ingredients, high-field EPR and PELDOR, pioneering work has been done in Moscow and Novosibirsk, respectively.

As a side note, it must have been frustrating for Jim Hyde and his science colleagues at Varian Associates in Palo Alto that such a large market for state-of-the-art EPR spectrometers was not going to be properly harvested by the Varian management in the 1970s, despite the growing scientific contacts between the scientists from Varian and the Soviet EPR laboratories. It gave some comfort when he convinced himself that for all these trips to exhibitions in the USSR …” the primary motivation, not only of Varian scientists and their Soviet colleagues, but also of Varian management (and possibly of Soviet leaders) was an improved world order” [10]. The frustration must have been even deeper when Varian Associates decided to drop out of the EPR business in the 1980s.

With regard to EPR in the Soviet Union of the 1970s, a real contrast program to Varian Associates was pursued by Bruker Physik-AG, then a young German enterprise making NMR and EPR spectrometers (later transformed into Bruker BioSpin Ltd, Rheinstetten, Germany): In the early 1970s the Bruker company opened the first office in USSR with Uwe Eichhoff  as representative—together with his charming wife Barbara. The two of them were highly recognized by the Soviet scientists and administrators. They established excellent relationships with the USSR Academy of Sciences and its follow-up Russian Academy and institutions, and many former Soviet universities. It is also their merit to have built up such a great reputation for Bruker and its EPR and NMR spectrometers, as well as MRI tomographs, in Russia and the CIS countries which has continued up to the present day [11].

Tragically, Voevodsky died suddenly on February 20, 1967, of a heart attack at the age of only 49 years. This tragedy happened 5 months before he had planned to open the eighth International Symposium on Free Radicals (July 26–31, 1967) in Novosibirsk. Heroically, Voevodsky’s co-workers took over the burden of organization, in particular Yuri Tsvetkov and Yuri Molin, and finally they opened the symposium in his memory.

It was at this 1967 Free Radical Conference in Novosibirsk when K.M. met Jim Hyde and Jack Freed for the second time, 2 years after their first encounter. In fact, at the Akademgorodok meeting, ENDOR was now among the strong topics of the conference program. Accordingly, both of them, Jim Hyde and Jack Freed, were appreciated as the great stars of ENDOR-in-solution, with deep admiration of what they had achieved together with August H. Maki at Harvard. Jack Freed had provided the theoretical basis for liquid-phase ENDOR with a series of keynote papers on saturation and relaxation of radicals in solution. Unfortunately, he got sick right at the beginning of the meeting and spent most of the time in hospital in Akademgorodok. Hence, Jim Hyde became the lonely ENDOR star, always surrounded by a flock of admirers, across all age groups.

It was still the time of the Vietnam War. And walking from the hotel to the conference hall over a railway bridge one could see endless cargo trains going South to Vietnam, loaded with tanks and cannons, hardly covered by canvas sheets. The American and Russian conference participants, together with their international colleagues, used to lean over the bridge railings watching the trains. Probably exchanging their thoughts and worries about their brothers and sons serving as soldiers or military advisors in Vietnam. Apparently, they understood each other by far better than one could have hoped considering the harsh official political statements at those times from both sides of the Iron Curtain.

And then, in the evening of the conference dinner, in the middle of the Siberian taiga around Akademgorodok, again Jim Hyde was the star of the evening. Vodka, and more vodka was flowing, helping to exchange old and new ideas, and to establish new East–West collaborations and friendships, tunneling the Iron Curtain. Some of them are lasting until today, for example Jim Hyde’s sustaining EPR connections to Novosibirsk, Kazan and Krakow. Notably, his EPR and MRI research has been carried out over the years with several staff engineers and scientists from the Jagiellonian University in Krakow, with his closest collaborator Wojciech Froncisz, working at the forefront of EPR innovations.

At both sides of the Atlantic there have been many more personal encounters with Jim Hyde to remember. But here we restrict ourselves to a very special meeting in Milwaukee in 2005 when K.M. followed the invitation to participate in the EPR Instrumentation Workshop, Milwaukee, May 6–7, 2005. The workshop was jointly organized by Jim Hyde from the National Biomedical EPR Center of MCW and Arthur Schweiger from the Physical Chemistry Laboratory at the ETH Zurich. Four plenary lectures were presented by Klaus Möbius (“High-field EPR and ENDOR at 95 and 360 GHz: instrumentation and biological applications”), James Hyde (“Resonator discoveries using finite element modeling of fields”), Arthur Schweiger (“EPR@ETH—Instrumentation and methodology, a historical outline”) and Wojciech Froncisz (“Digital EPR transceiver with high speed field programmable gate array”). In addition, keynote lectures were presented by Richard Mett (Medical College of Wisconsin), Rolf Schuhmann (Technical University Darmstadt, Germany), Boris Epel (Max-Planck-Institute for Bioinorganic Chemistry, Mülheim (Ruhr), Germany), Jörg Forrer (ETH Zurich, Switzerland), Igor Gromov (ETH Zurich, Switzerland), Robert Strangeway (Medical College of Wisconsin), Candice Klug (Medical College of Wisconsin).

**Fig. 3:**
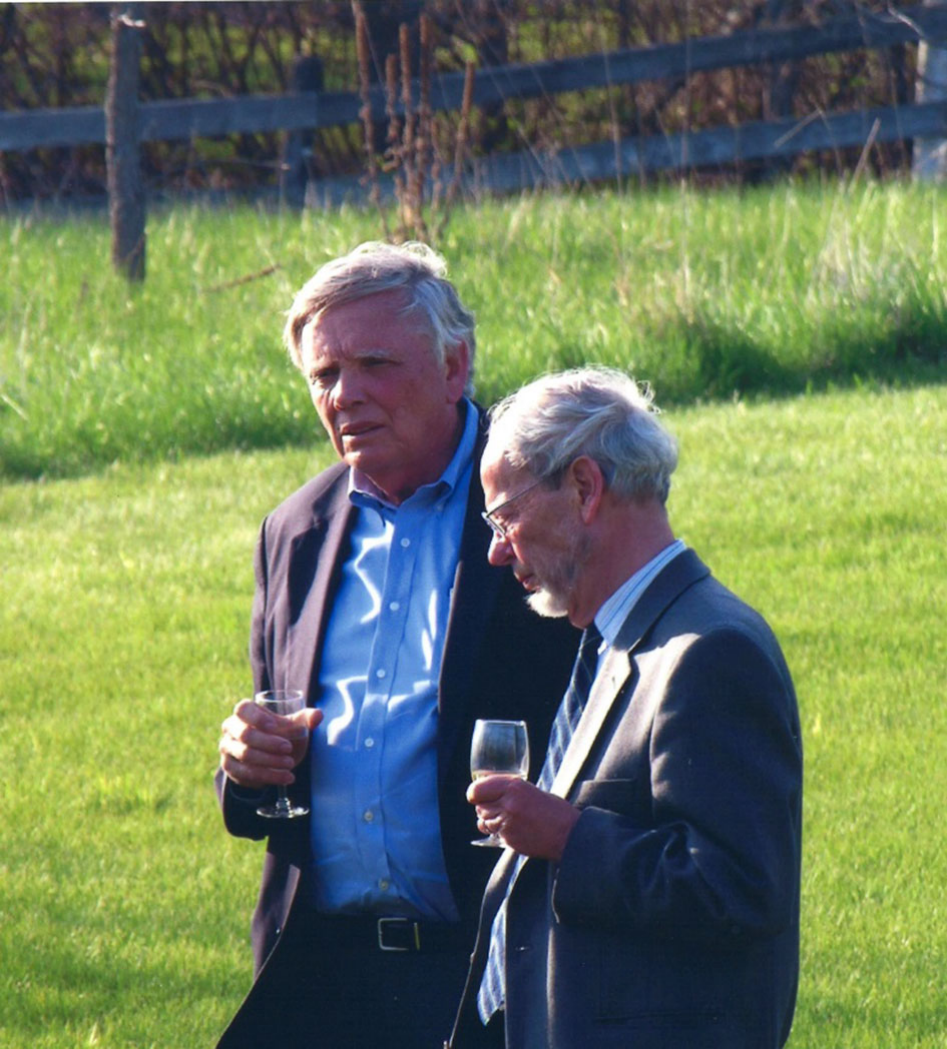
Jim Hyde and Klaus Möbius (2005) in the garden of the Hyde house in Milwaukee (photo: Arthur Schweiger)

Jim Hyde and his wife Karen were kind enough to take the foreign speakers to their house in the marvelous Wisconsin countryside where we could admire not only their agricultural instrumentation in the barn and corresponding expertise of farming, their fine art collection of paintings and prints from Wisconsin artists and European expressionists in the house, but also Jim Hyde’s extraordinary wine cellar, temperature-controlled, well-sorted and well-stocked. As seen in Fig. 3, serious matter can best be discussed over a glass of wine, even outdoor in the vast stretch of Hyde’s garden.

Next day, late in the afternoon, Jim Hyde invited K.M. for a visit of the Milwaukee Art Museum. It would be an architectural landmark, designed by legendary architects, among them Santiago Calatrava. The museum was already closed. But Jim Hyde, apparently as a prominent sponsor, had open access, also after business hours. Slightly bewildering, he took a straight way to the museum’s small, but superb collection of Gabriele Münter, the German expressionist of the early twentieth century. It was one of his favorite collections of the Milwaukee Art Museum, he said, and his crisply comments on the exhibits were a delight to listen.

**Fig. 4:**
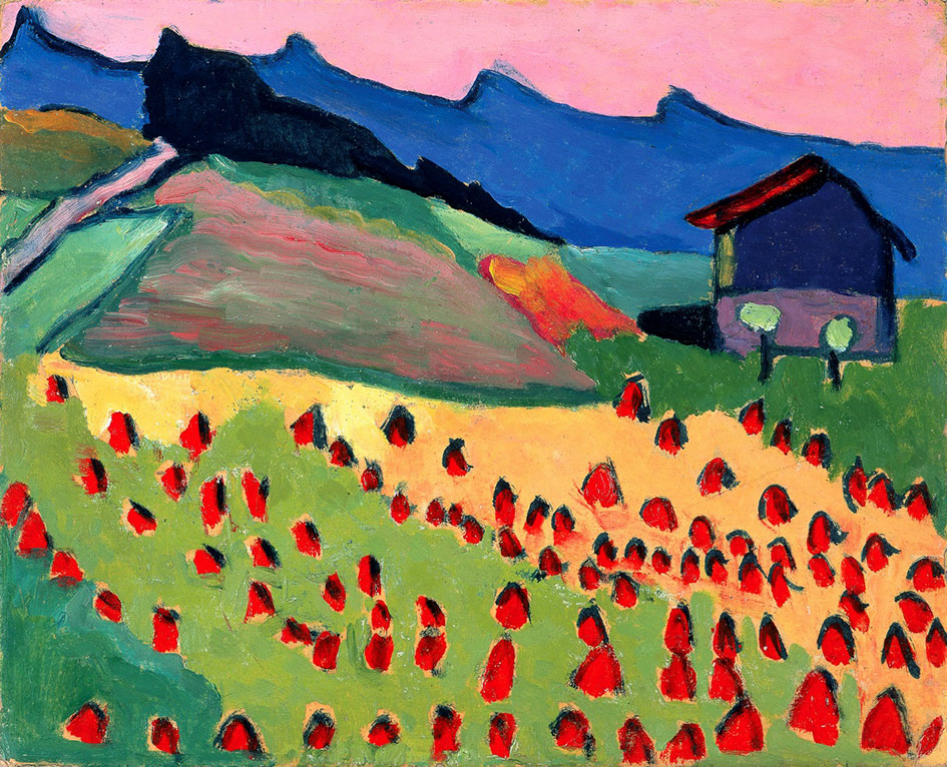
Gabriele Münter (1877–1962), Landschaft mit Hütte im Abendrot (Landscape with cottage in the sunset glow), 1908, Museum Gunzenhauser, Chemnitz

Now, we are left with our congratulations to Jim Hyde’s 85th birthday on May 2, 2017. Our encounters with you over the years were marked by exciting science (very important) and inspiring personal interactions (not less important). For this we want to thank you, Jim, adding a small gift as a sign of affection (Fig. 4).

Again, Dear Jim, congratulations on your birthday! Best wishes for good health and happiness, and lots of success with your next ideas and projects!

### Selected ENDOR and TRIPLE Experiments in Liquid Solution

Now, we continue with a few examples of our own work: at first ENDOR and TRIPLE resonance in liquid solution, hereafter ENDOR, EDNMR, and PELDOR in frozen solution. The papers selected for this *Festschrift* start in 1975 with our joint X-band ENDOR-in-solution experiments together with Jim Hyde on low-symmetry radical anions of phenylcyclazines [12]. Noticeably, this cooperation took not less than 10 years to happen after our first encounter in Cirencester.

### ENDOR in Solution

There are many good reasons for physicists, chemists and biologists to celebrate the happy liaison between EPR and NMR with ENDOR as the first offspring, born in 1956 in the solid state at low temperature; George Feher was the matchmaker and happy father [13]. And Jim Hyde and Gus Maki were among his highly gifted followers, trying hard—and ultimately successful—to develop ENDOR in liquids at elevated temperatures [14]. The main motivation for extending single-resonance EPR to double-resonance ENDOR techniques is twofold: (1) to enhance the sensitivity of detection by “quantum transformation” from the low-frequency NMR domain, where the radiofrequency (rf) transitions occur to be measured, to the high-frequency EPR domain, where spectral changes due to the absorbed microwave (mw) energy are detected, and (2) to enhance the resolution of the spectrum, i.e., to reduce the number of spectral lines in a given frequency range by imposing both EPR and NMR “selection rules” on the induced transitions. Thereby, redundant hyperfine lines are eliminated in the inhomogeneously broadened EPR spectrum. As a result, the line density in an ENDOR spectrum increases only in an additive way with increasing number of groups of equivalent nuclei, whereas in an EPR spectrum it increases in a multiplicative way.

George Feher had coined the name ENDOR that evidently stands for “electron–nuclear double resonance”. Interestingly, Feher had been inspired for choosing this acronym also by the Old Testament [15]: King Saul of the Kingdom of Israel did the unthinkable when menacingly asking the Witch (a magician woman) of Endor (a village south of Mt. Tabor near the Sea of Galilee) to make visible the invisible and to tell him the untellable: What will be his fate next day in the battle against the assembled forces of the Philistines (see The Old Testament, Samuel I, Chapter 28:7). Although the witch’s courage faltered she tried hard to obey King Saul’s order and call back the shadows from the underworld. And she succeeded but received bad news to tell the king: King Saul and his army will be defeated in the battle, and Saul himself will be among the multitude of killed soldiers. In contrast, George Feher’s ENDOR experiment of making visible the invisible hyperfine couplings, brought him good news when mysteriously out of the inhomogeneously broadened totally unresolved EPR spectrum several narrow well-resolved hyperfine lines appeared—very much like one would imagine to see in an NMR spectrum of the paramagnetic sample.

Under continuous wave (cw) mw and rf irradiation, as common for liquid samples to which we restrict ourselves in the moment) ENDOR signals are obtained by monitoring the changes of the amplitude of a saturated EPR line when sweeping a saturating rf field through the NMR frequency region. In solid-state samples the electron and nuclear relaxation times are sufficiently long to easily obtain saturation. For doublet-state radicals in liquid solution, however, the relaxation times are much shorter—in the order of 10^−5^ to 10^−7^ s—and, consequently, much larger saturating mw and rf fields are needed. This probably explains why it took another 8 years since Feher’s first ENDOR experiment on phosphorus-doped silicon at low temperature [13] before the first ENDOR-in-solution experiments on organic radicals could be successfully performed by Hyde and Maki [14].

To learn from the experience Gus Maki had accumulated already on ENDOR in solution at Harvard and UC Riverside, K.M. spent a postdoctoral year 1969/1970 in his laboratory. He had hoped to benefit from it for his group’s own high-power ENDOR efforts at FU Berlin which were still in the early stages of development. In 1966, he had successfully applied for a DFG grant to start an ENDOR-in-solution project with high-power mw and rf irradiation sources, with dedicated mw cavities and a tuned-circuit rf generator. In the Maki lab in Riverside K.M. met Hans van Willigen, a postdoc from the University of Nijmegen. Hans also had come to Riverside to do ENDOR experiments on organic radicals in solution. On the premises, our work plans had to be changed drastically from what we had expected. Instead of recording nice ENDOR spectra in due time, we shared the frustration—and fun—of day-and-night efforts to rebuild the widely dismantled ENDOR spectrometer; it had been cannibalized after Robert Allendoerfer had left the Maki group for the State University of New York at Buffalo.

Ultimately, we found a stable solution to the problem of devastating stray fields by impedance matching the high rf power amplifier to the ENDOR coil by incorporating an (empty) California wine bottle wrapped with a few turns of heavy copper wire (see [16]). This resort to empty (and full) California wine bottles enabled us to jointly perform an ENDOR study on the lifting of orbital degeneracy in large high-symmetry molecules by introducing weak methyl-group perturbations. We chose pentaphenyl-cyclopentadienyl (PPCPD) successively methyl-substituted at the para positions [17]. The samples were a generous gift by our chemistry friend Harry Kurreck from FU Berlin.

For several years thereafter, only a few groups around the world invested the time (and grant money) to build their own ENDOR-in-solution spectrometers with high-power rf capability. When commercial ENDOR spectrometers became available in the mid-1970s [10, 11] the field exploded with numerous applications from chemistry, biochemistry and molecular physics (for overviews, see [18–29]).

The development and understanding of ENDOR-in-solution spectroscopy was highly stimulated by Jack Freed [30–32] and his work on spin relaxation. His general theory of EPR saturation and double resonance in liquids proved to be highly adequate in describing amplitude, width and shape of ENDOR lines in great detail, including subtle coherence effects due to the strong mw and rf fields [32].

One of these specific coherence effects is particularly interesting because it can be exploited for assigning ENDOR lines to molecular positions [32], which is a crucial task when applying ENDOR as an analytical tool for identifying unknown radical species. This coherence phenomenon causes exploitable line shape effects such as specific line broadening or line splittings in the ENDOR spectrum, and requires nuclear spins *I* > 1/2 or a set of at least two equivalent nuclei of *I* = 1/2. The magnitude of the coherence splitting is dependent on the number of nuclei involved, the hyperfine transitions being mw saturated, and the rf field strength driving the NMR transitions of the spin system. In 1971, such a coherence effect was exploited by Klaus-Peter Dinse in the Möbius group at FU Berlin during his PhD work [33–35] to assign hyperfine splittings in the ENDOR-in-solution spectra of various low-symmetry radicals by counting the number of protons contributing to a specific ENDOR line. A dedicated cylindrical ENDOR cavity (TE_011_ mode) was constructed to achieve sufficiently strong cw rf fields up to 3 mT (rotating frame). The internal NMR coil is part of the power stage of a 1 kW cw rf transmitter station. To secure thermal stability of the cavity frequency, effective water cooling is employed both for the cavity body and the two-loop NMR coil [35, 36].

Since ENDOR is inherently a variant of NMR for paramagnetic systems, the unpaired electron serving as highly sensitive detector for the NMR transitions Δ*m*_*Ii*_ = ± 1, each group of equivalent nuclei—no matter how many nuclei are involved and regardless of the value of their individual spin—contributes only two ENDOR lines to the spectrum. The two lines of a particular group of equivalent nuclei appear at:

1 $${\nu_{\text{iENDOR}}^{ \pm } = |\nu_{n} \pm A_{i} / 2|}.$$

Here, the nuclear Larmor frequency is given by *ν*_*n*_ = *g*_*n*_ *μ*_K_ *B*_0_/*h*, and the hyperfine coupling parameter A contains isotropic and anisotropic contributions of the hyperfine tensor ${\tilde{A}}$. In isotropic solution, the hyperfine couplings (hfcs) are given by *A*_iso_ = 1/3 Tr(${\tilde{A}}$), the Fermi contact interaction parameters. Hence, in ENDOR with each set of inequivalent nuclei the number of resonance lines in the spectrum increases merely in an additive way.

**Fig. 5:**
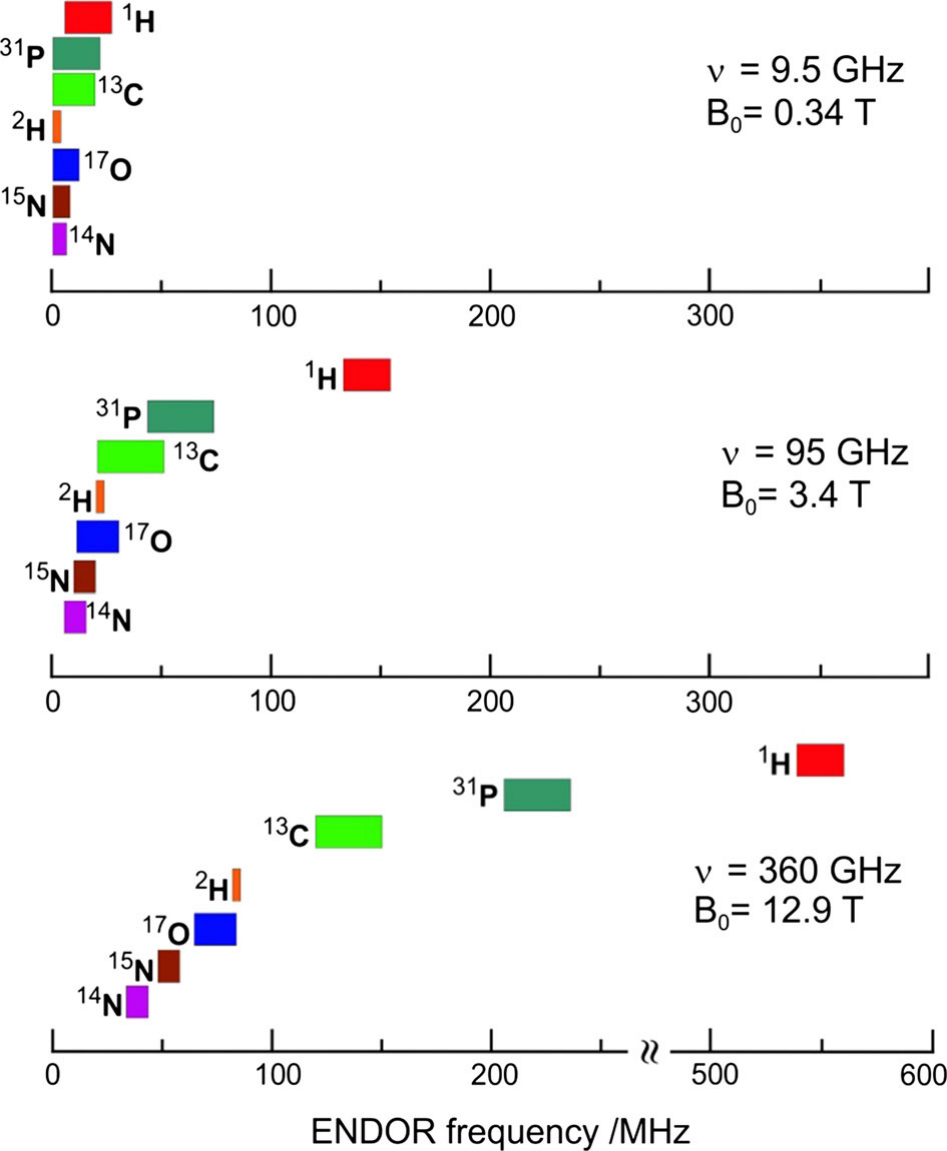
Improved ENDOR resolution for different nuclei in doublet-state systems (*S* = 1/2, *g* = 2) with increasing mw frequencies and Zeeman fields

Apparently, the gain in resolution of ENDOR versus EPR becomes most significant for low-symmetry molecules with a large number of groups of symmetry-related nuclei. The resolution enhancement is particularly drastic when nuclei with different magnetic moments are involved. Their ENDOR lines appear in different frequency ranges and, providing that their Larmor frequencies are separated at the chosen Zeeman field B_0_, the different nuclei can be immediately identified. In the case of an accidental overlap of ENDOR lines from different nuclei at X-band (9.5 GHz and 0.34 T) the lines can be separated at higher Zeeman fields and microwave frequencies, for instance at 3.4 T and 95 GHz [37] or even at 12.9 T and 360 GHz [38]. The disentangling of ENDOR lines at different fields is depicted in Fig. 5. In biological molecules containing several magnetic nuclei other than protons, the separation of accidentally overlapping ENDOR lines is extremely helpful for analyzing complex spin systems by means of their nuclear Zeeman and hyperfine interactions.

**Fig. 6:**
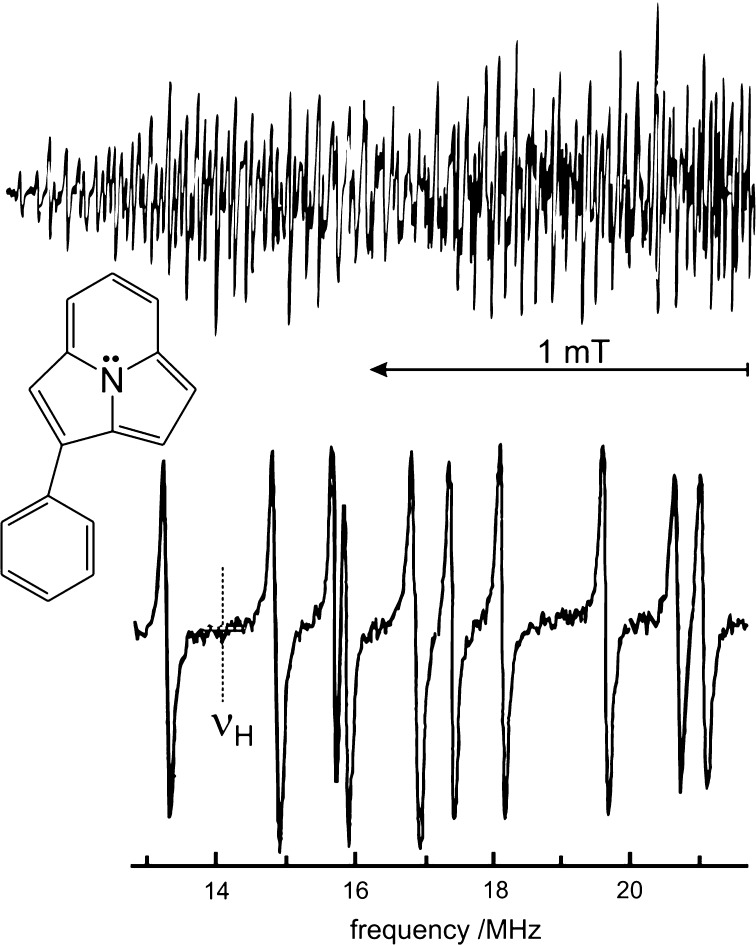
(Top) High-field half of the EPR spectrum of the 2-phenyl[3.2.2]azine radical anion (solvent: DME, *T* = 210 K). (Bottom) High-frequency half of the “high-power” proton ENDOR spectrum (*T* = 180 K) Adapted from [12]

An illustrative example of the power of liquid-phase ENDOR is given in Fig. 6 for the phenylcyclazin radical anion in solution [12]. Six co-authors from three laboratories (F. Gerson, University of Basel; K. Möbius, Free University Berlin; J.S. Hyde, Varian Ass., Palo Alto) were involved and, indeed, the ENDOR resolution achieved was just stunning and worth the combined efforts. In this case the ratio of the spectral densities of EPR and ENDOR is ca. 30. Since the widths of the EPR and ENDOR lines are similar, this factor is fully gained as a bonus in resolution.

The proton hyperfine couplings have been assigned by a combination of four methods: rf coherence effects, calculations of relative ENDOR intensities, computer simulations of the EPR spectra, and MO calculations of π-spin distribution over the entire molecule. The successful use of the two first methods has experimentally corroborated Jack Freed’s theory of ENDOR line-shapes in the presence of both saturating and non-saturating nuclear radio frequency fields [30–32].

The example of the phenylcyclazin radical anion in solution clearly shows already that in cw ENDOR the bonus of increased spectral resolution has to be paid off by inherent drawbacks of the method: ENDOR signals reach only some percent of the EPR signal, which means that one loses dramatically in sensitivity. The intensity of the ENDOR-in-solution effect has to be maximized by carefully controlling temperature and viscosity of the solvents, thereby optimizing the delicate interplay between electron and nuclear relaxation rates. In the ideal case, they should be made equal. In addition, as is obvious from Fig. 6, the ENDOR line intensities are not proportional to the number of contributing nuclei, while in EPR, the intensity pattern directly reflects the number of nuclei involved in the spin transitions.

These drawbacks can be overcome by extending ENDOR to electron–nuclear–nuclear triple resonance (TRIPLE) as was proposed by Jack Freed [39] in 1969 and experimentally realized 1974/75 by K.P. Dinse and R. Biehl in the Möbius group at FU Berlin [3, 18, 40] (see below).

On the basis of Freed’s relaxation theory for radicals in fluid solution [30–32], Martin Plato, Wolfgang Lubitz and co-workers in the Möbius group [20, 41–44] carried out a systematic investigation of the ENDOR sensitivity of various hetero-nuclei, i.e., nuclei other than protons, in organic radicals. Optimum ENDOR conditions, such as temperature and viscosity of the solvent, mw and rf field strengths, were formulated by employing the rigorous density matrix formalism as a function of a few nuclear and molecular properties [41]. They include relaxation from fluctuating spin–rotation interaction, electron–nuclear dipolar and nuclear quadrupolar couplings and Heisenberg spin exchange. The most important molecular parameter turned out to be the magnitude of the anisotropy of electron-nuclear dipolar interaction. The theoretical results were found to be in good agreement with experimental observations on ^2^H, ^13^C, ^14/15^N, ^19^F, ^31^P and alkali nuclei in different molecular systems, thus allowing predictions to be made on the ENDOR detectability of other chemically interesting nuclei, such as ^10/11^B, ^17^O, ^27^Al, ^29^Si, ^33^S and ^35/37^Cl. In the meantime, most of these nuclei have indeed been detected by ENDOR in solution [22, 28]. In biological molecules, often several magnetic non-proton nuclei are present, and at X-band (9.5 GHz, 0.34 T) their ENDOR lines may overlap accidentally. As can be seen in Fig. 5, they become separated by working at higher mw frequencies and corresponding Zeeman fields, for instance at 95 GHz, 3.4 T or even at 360 GHz, 12.9 T.

### TRIPLE Resonance as an Extension of ENDOR in Solution

In the frequently occurring cases where electron–nuclear cross-relaxation with flip–flop rate *W*_*x*1_ and flop–flop rate *W*_*x*2_ does not operate, e.g., at lower temperatures—and thus cannot increase the ENDOR enhancement—maximum ENDOR-in-solution signals are obtained when the “matching condition” for the electron and nuclear relaxation rates, *W*_e_ = *W*_n_, is fulfilled [41]. Such a matching condition is often difficult to meet for a specific system when one tries to select the proper solvent, temperature and viscosity. This is particularly true for biological systems at physiological temperatures for which *W*_n_ ≪ *W*_e_ is the common situation. As a consequence, for *W*_*x*1_ = *W*_*x*2_ = 0, the slow *W*_n_ acts like a bottle-neck for the rf-induced EPR desaturation, thereby drastically reducing the ENDOR signal intensity.

**Fig. 7:**
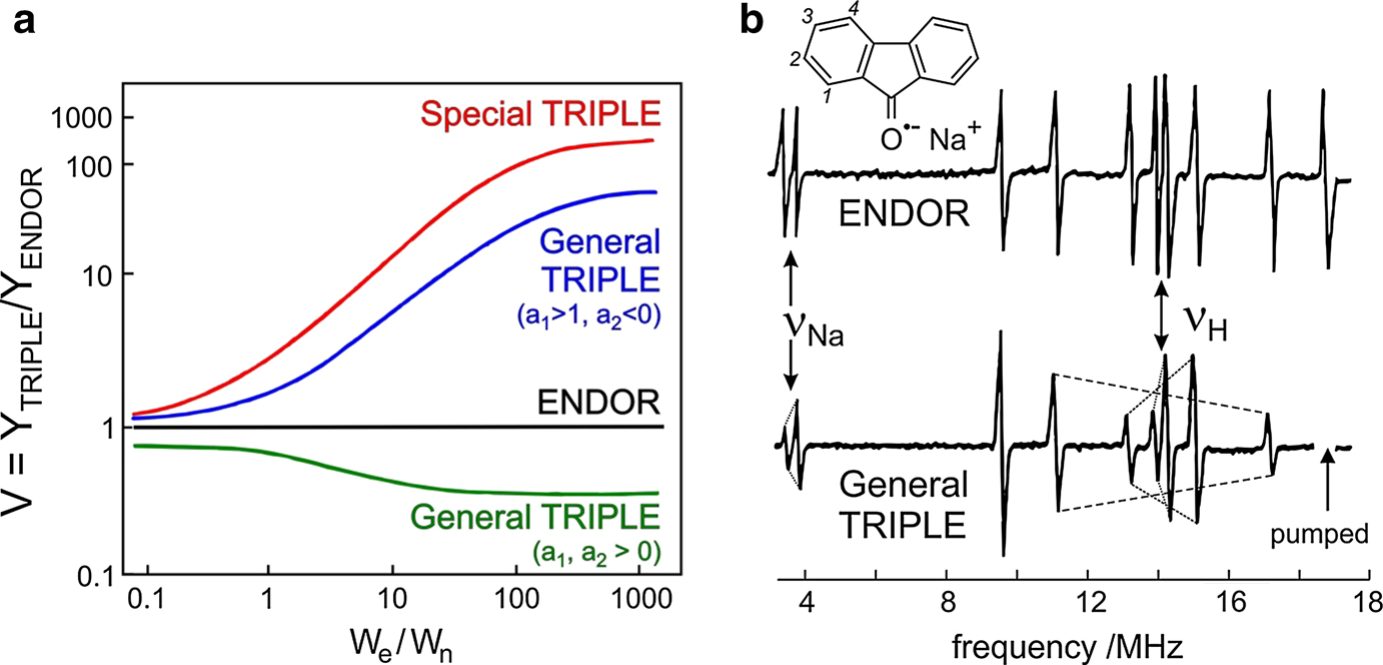
**a** TRIPLE amplification factor (ratio of TRIPLE and ENDOR line amplitudes) as function of *W*_e_/*W*_n_, **b** ENDOR and general TRIPLE spectra of the radical anion fluorenone^−·^ (solvent: tetrahydrofuran, counter ion: Na^+^, *T* = 226 K). Adapted from Ref. [18]. Note the different signs of the hyperfine couplings of Na^+^ around *ν*_Na_ and protons around *ν*_H_. For details see [18, 44, 47]

There is an obvious solution to this problem by “short-circuiting” the *W*_n_ bottle-neck, i.e., by applying two rf fields tuned to drive both NMR transitions, *ν*^+^ and *ν*^−^, of the same nucleus; see Eq. (1). Such a special electron–nuclear–nuclear triple resonance was proposed by G. Feher [45] and J.H. Freed [39], but was first experimentally realized for a radical in liquid solution by K.P. Dinse and co-workers [40] (“Special TRIPLE” [18]). As was demonstrated by R. Biehl and co-workers [3], additional information about relative signs of hyperfine couplings of radicals in solution can be obtained by generalizing the triple-resonance experiment to include NMR transitions of different nuclei in the radical (“General TRIPLE” [18]). The analog of this experiment for solid-state samples at low temperature was performed earlier by R.J. Cook and D.H. Whiffen [46]. The advantages of TRIPLE over ENDOR—enhanced sensitivity and resolution, information about multiplicity and relative signs of hyperfine couplings from line intensity variations are become apparent from Fig. 7a where the TRIPLE amplification factors V are plotted versus *W*_e_/*W*_n_. In the common case *W*_e_/*W*_n_ ≫ 1, Special TRIPLE can approach 100% EPR sensitivity, and different relative signs of hyperfine couplings are reflected by amplitude changes of the General TRIPLE lines. Figure 7b gives an experimental verification of this analysis [43, 47].

**Fig. 8:**
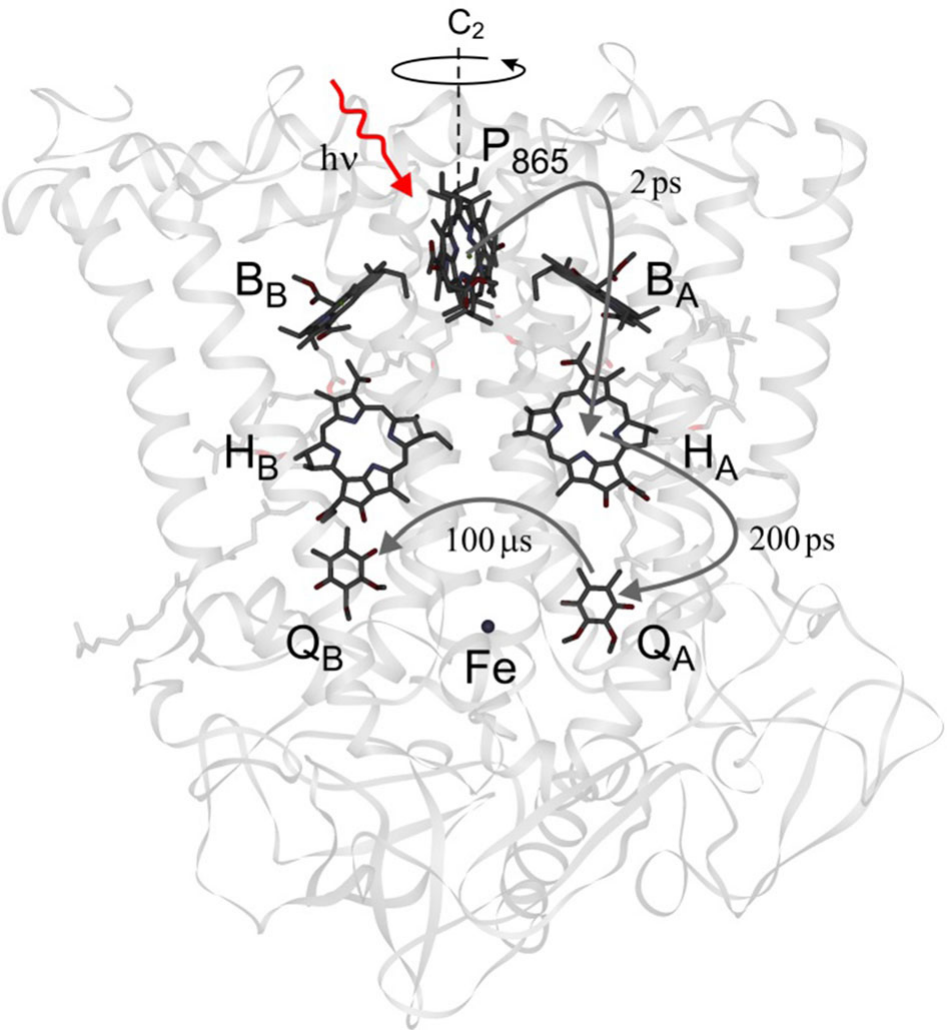
X-ray structural model of the RC from the carotinoid-less strain R26 of *Rhodobacter* (*Rb.*) *sphaeroides* [50]. The RC contains nine cofactors: the primary donor P_865_ “special pair” [a bacteriochlorophyll *a* (BChl) dimer], two accessory BChls (B_A_, B_B_), two bacteriopheophytins *a* (BPhe: H_A_, H_B_), two ubiquinones (Q_A_, Q_B_) arranged in 2 branches (A, B), one non-heme iron (Fe^2+^). Light-induced electron transfer proceeds exclusively along the A-branch of the protein-embedded cofactors despite the approximate C_2_ symmetry of the cofactor arrangement. The time constants of different charge separation and transfer events are indicated. In the samples studied by EPR the high-spin Fe^2+^ (*S* = 2) has been replaced by Zn^2+^ (*S* = 0). For details, see references given in the text

The TRIPLE-resonance techniques were shown later to be extremely powerful in elucidating the electronic structures not only of organic radicals in solution [22], but also of transient cofactor radical ion intermediates in the reaction center protein complex of primary photosynthesis [25, 29, 48].

### ENDOR/TRIPLE on Primary Donor Radical Cations  in Bacterial Photosynthesis

At this point it is appropriate to remember late Arnold Hoff (University of Leiden) whom K.M. first met during an EPR symposium in Nijmegen, in sweltering August of 1976. Over a glass of cool beer or two Arnold introduced him to the beauties of photosynthesis, and we discussed joint ENDOR-in-solution experiments on the electron transfer cofactors in bacterial photosynthesis. During his first—unforgettable—visit to snowy pre-Christmas Berlin in 1977, nitrogen ENDOR and proton TRIPLE spectra of the bacteriochlorophyll *a* cation radicals in fluid organic solvents could be recorded after tiring attempts to optimize temperature and concentration. Finally, most of the hyperfine couplings (including their signs) were measured. The results were jointly published in PNAS in May 1978, they had been communicated by George Feher in February 1978 [49]. It is very sad that Arnold Hoff died so early, in 2002 at the age of 63.

In the following, we review an acid test of the ENDOR-in-solution methodology for resolving complex hyperfine structures of extremely large molecules: ENDOR experiments on the radical cation of the primary donor dimer P_865_, the “special pair” of bacteriochlorophylls (BChl). These molecules are redox cofactors in the photosynthetic electron transfer in the reaction center (RC) of purple bacteria *Rb. sphaeroides* [51]. The ENDOR-in-solution work on such RCs at physiological temperatures was a cooperation project with Hugo Scheer and his group at the Botanical Institute of the University of Munich [52–55]. Thanks to his superb RC preparations liquid-phase ENDOR became feasible. Later, George Feher and his group at UCSD also contributed outstanding mutant and wild-type RC preparations to the joint ENDOR and TRIPLE-resonance work [28, 29, 54, 56].

**Fig. 9:**
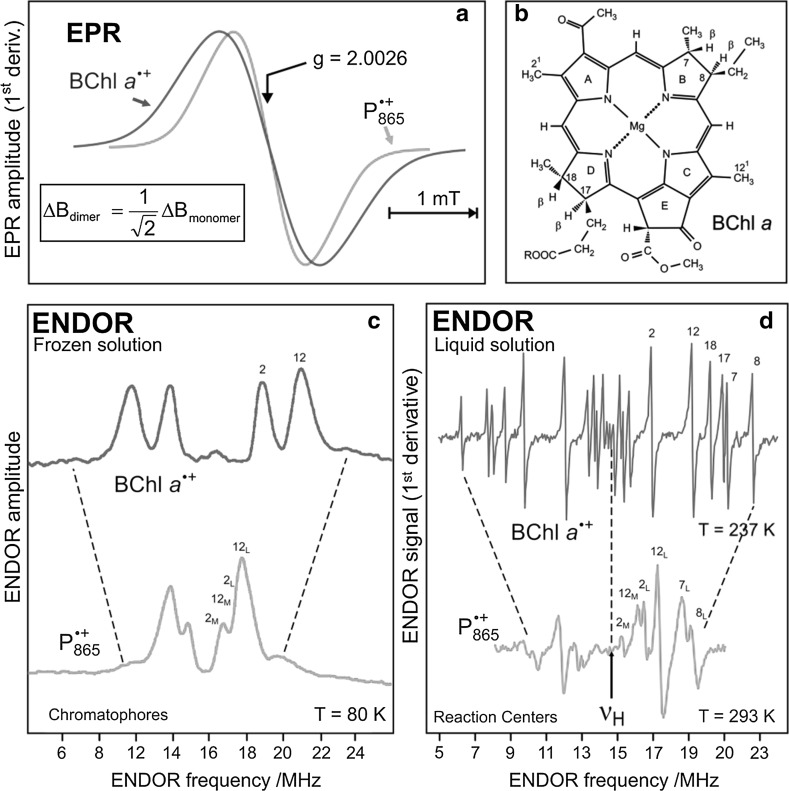
**a** EPR spectra (X-band) at ambient temperature of the radical cations of BChl *a* in organic solution and of P in the RC. Both have the same *g* value and saturation behavior but the linewidth of ${{\text{P}}_{ 8 6 5}^{ \cdot + } }$ is approx. 1/${\sqrt 2}$ narrower than that of ${{\text{BChl}}\,a^{ \cdot + } }$ indicating a dimeric species. **b** Molecular structure of BChl *a* with numbering scheme. **c** Comparison of the ^1^H ENDOR spectra of ${{\text{BChl}}\,a^{ \cdot + } }$ and ${{\text{P}}_{ 8 6 5}^{ \cdot + } }$ in frozen solution (80 K). Two strong resonances of the methyl protons (pos. 2 and 12) are visible; the respective hyperfine couplings are reduced in ${{\text{P}}_{ 8 6 5}^{ \cdot + } }$ indicating spin delocalization in the BChl *a* dimer. **d** Comparison of high-resolution ^1^H ENDOR spectra of ${{\text{BChl}}\,a^{ \cdot + } }$ and ${{\text{P}}_{ 8 6 5}^{ \cdot + } }$ in isotropic liquid solution. From the assigned individual isotropic hyperfine couplings a detailed picture of the spin density distribution in the monomeric ${{\text{BChl}}\,a^{ \cdot + } }$ and in the dimer ${{\text{P}}_{ 8 6 5}^{ \cdot + } }$ is obtained [52–54, 63, 64]

The primary processes of photosynthesis provide a “Garden of Eden’’ for the EPR spectroscopists [57] because in each electron-transfer step a transient paramagnetic intermediate is formed. In this overview, a brief account of such studies is presented that have led to the identification and characterization of the primary ion radicals of the electron-transfer chain containing, for example, the P_865_ cation, and also bacteriopheophytin anion and the ubiquinone anion radicals. The comparison of the frozen-solution EPR spectra of monomeric ${{\text{BChl}}\,a^{ \cdot + } }$ in an organic solvent and ${{\text{P}}_{ 8 6 5}^{ \cdot + } }$ in the RC revealed a striking difference in the linewidth of both EPR spectra: for ${{\text{P}}_{ 8 6 5}^{ \cdot + } }$ it is 1/ ${\sqrt 2}$ narrower than for ${{\text{BChl}}\,a^{ \cdot + } }$; see Fig. 9a. J.R. Norris, J.J. Katz and co-workers [58] explained this observation by the ‘‘special pair’’ hypothesis, i.e., the unpaired electron of ${{\text{P}}_{ 8 6 5}^{ \cdot + } }$ is equally shared between the two halves of a (BChl *a*)_2_ dimer cation radical, which they assumed to be symmetric. G. Feher, A.J. Hoff and co-workers [59, 60] confirmed the dimeric nature of ${{\text{P}}_{ 8 6 5}^{ \cdot + } }$ by comparing the ENDOR spectra of frozen-solution samples of ${{\text{BChl}}\,a^{ \cdot + } }$ and ${{\text{P}}_{ 8 6 5}^{ \cdot + } }$. Similar ENDOR experiments were performed independently around the same time by J.R. Norris et al. [61, 62]. The ENDOR spectra showed that the few resolved hyperfine couplings (marked in Fig. 9c) are indeed approximately halved in ${{\text{P}}_{ 8 6 5}^{ \cdot + } }$, at least within the limits of the broad solid-state ENDOR lines of the experiments. This interpretation was later refined for RCs in fluid solution under physiological conditions by taking advantage of the high spectral resolution of ENDOR-in-solution [52–55, 63–65].

**Fig. 10:**
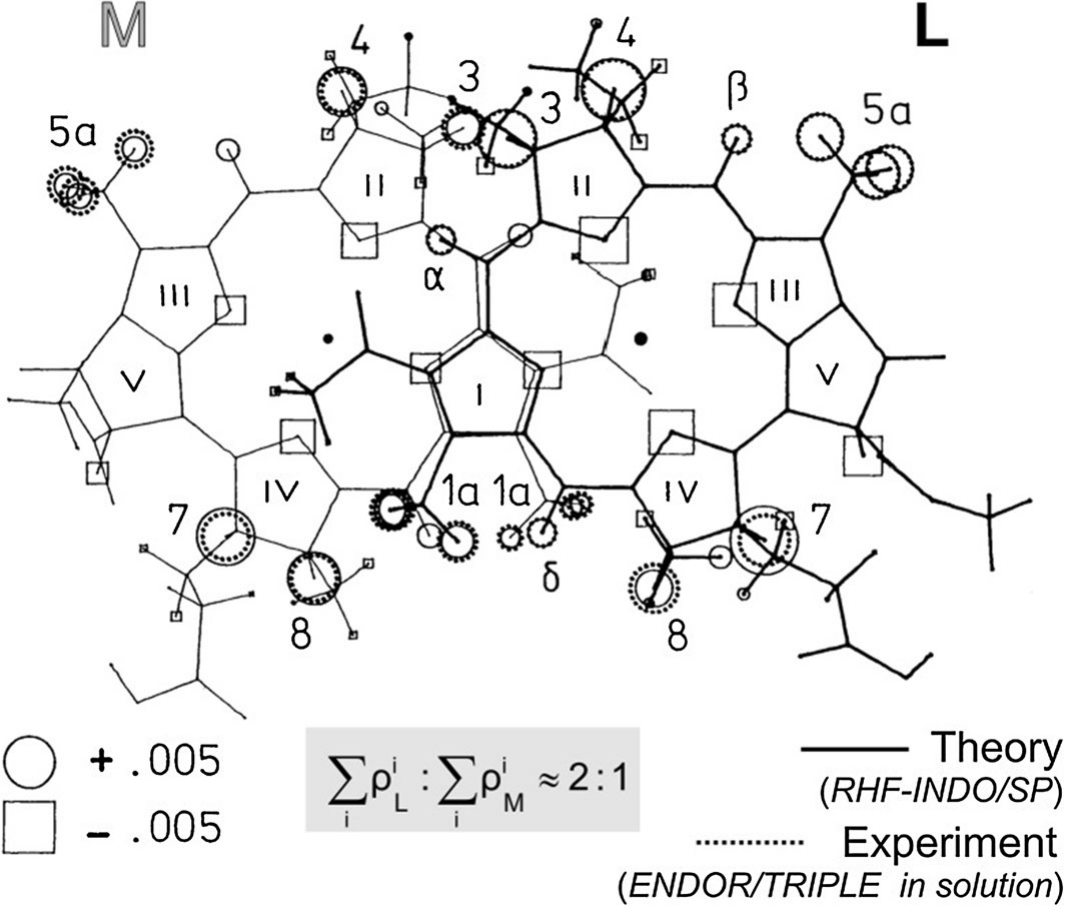
Comparison of experimental (dotted lines) and calculated (RHF-INDO/SP) [64] s-spin densities *ρ*_H_(1s) (solid lines) of ${{\text{P}}_{ 8 6 5}^{ \cdot + } }$ in *Rb. sphaeroides*. Experimental values from isotropic proton hyperfine couplings using *A*_iso_ = *Q*_H_·*ρ*_H_(1s) with *Q*_H_ = 1420 MHz [55]. Geometry from X-ray structure analysis [50]. The s-spin densities are proportional to the areas of the respective squares (*ρ* < 0) and circles (*ρ* > 0). For rotating methyl protons, the average value of the three proton s-spin densities is shown. For details of the experiments and calculations, see Refs. [55, 64]

To resolve additional hyperfine couplings for a more thorough comparison of ${{\text{BChl}}\,a^{ \cdot + } }$ and ${{\text{P}}_{ 8 6 5}^{ \cdot + } }$, K. Möbius, F. Lendzian, W. Lubitz and their co-workers [52–54, 63, 64] applied ENDOR and electron–nuclear TRIPLE resonance to fluid-solution samples under physiological conditions exhibiting intrinsically narrower lines. At physiological temperatures the monomeric, detergent-solubilized RCs are tumbling fast enough to average out the anisotropic *g* and hyperfine contributions, resulting in highly resolved ENDOR-in-solution spectra (see Fig. 9d). From the highly resolved hyperfine structures of the monomers and dimers and their analysis by all-valence electron MO methods, notably by RHF-INDO/SP method developed by M. Plato, see [64], it was concluded that (1) the primary donor dimer has to be viewed as a supermolecule with the wavefunction extending over both dimer halves; (2) the symmetry of the electron spin density distribution over the two dimer halves is broken favoring the L half in a ratio 2:1 on the average, thereby manifesting that the ‘‘special pair’’ is electronically an asymmetric dimer. This finding was corroborated by comparing the experimental spin densities of ${{\text{P}}_{ 8 6 5}^{ \cdot + } }$ with theoretical predictions based on state-of-the-art quantum-chemical calculations [64] (see Fig. 10).

The final assignments of measured hyperfine couplings to molecular positions resulted from specific deuterations and ^15^N labeling [48, 65] as well as from investigations of ${{\text{P}}_{ 8 6 5}^{ \cdot + } }$ in RC single crystals [55]. The analysis shows an asymmetric spin distribution in favor of P_A_, the BChl dimer half bound to the L subunit (*ρ*_A_:*ρ*_B_ ≈ 2:1).

The single-crystal ENDOR and TRIPLE work on *Rb. sphaeroides* RCs at physiological temperatures, which were performed independently by the three groups of G. Feher at UC San Diego, W. Lubitz at TU Berlin and K. Möbius at FU Berlin, but ultimately published jointly [55], represents a culmination of two decades of EPR work on the primary donor in bacterial RCs [24]. For the first time it was possible to assign ENDOR lines unambiguously to the individual dimer halves of the primary donor cation. This work on the electronic structure of the primary donor in bacterial photosynthesis formed the basis for a large number of further studies on this species; see for example [66–72]. It has been discussed that such an asymmetry in the electronic structure of ${{\text{P}}_{ 8 6 5}^{ \cdot + } }$ might represent an important functional factor in controlling the vectorial properties of photosynthetic electron transfer to achieve a high quantum yield.

### ENDOR in Liquid Crystals

Evidently, magnetic-resonance spectroscopy in liquids excels by narrow lines, but sacrifices information on anisotropic interactions as long as isotropic solvents are used. This is because the anisotropic parts of tensor interactions are averaged out by rapid Brownian tumbling. However, by using liquid crystals as anisotropic solvents, valuable information about anisotropic interactions can be retrieved from line positions while retaining narrow hyperfine lines typical for liquid-solution spectra. In the nematic mesophase of a liquid crystal, solute molecules can be partially aligned in the external Zeeman field of an EPR spectrometer. This results, for axial symmetry of either the interaction or ordering tensor, in a shift of the measured interaction parameter relative to its isotropic value, *F*—*F*_iso_ = *O*_33_ $F_{33}^{\prime }$.

Here, *O*_33_ is the temperature-dependent ordering parameter, and $F_{33}^{\prime }$ is the principal component of the traceless interaction tensor that refers to the axis of highest symmetry of the solute molecule. *F* stands for any second-rank interaction tensor, for example the *g*-, hyperfine or quadrupole tensors.

The most striking aspect of ENDOR in liquid crystals is the possibility to directly determine, for nuclei with *I* > 1/2, components of the quadrupole interaction tensor of radicals in fluid solution from their ENDOR line positions. EPR in liquid crystals is not suitable in this respect because, to first order, the quadrupole interaction shifts all EPR-connected levels equally. The first determination of ^14^N quadrupole couplings in an organic radical was achieved by ENDOR in liquid crystals by K.P. Dinse et al. [73]. When cooling the liquid crystal from its isotropic to its nematic phase one observes shifts or even splittings of the ENDOR lines of the quadrupole nucleus (e.g., *I* = 1), depending on which EPR line (*M*_*I*_ = + 1, 0, −1) is saturated. The quadrupole splitting is given by:

2 $$\delta \nu_{\text{Q}} = ( 3/ 2){\text{O}}_{zz} e^{ 2} q_{zz} Q/h ,$$

from which *e*^2^*q*_*zz*_ *Q* can be deduced when the ordering parameter *O*_*zz*_ is known [74].

**Fig. 11:**
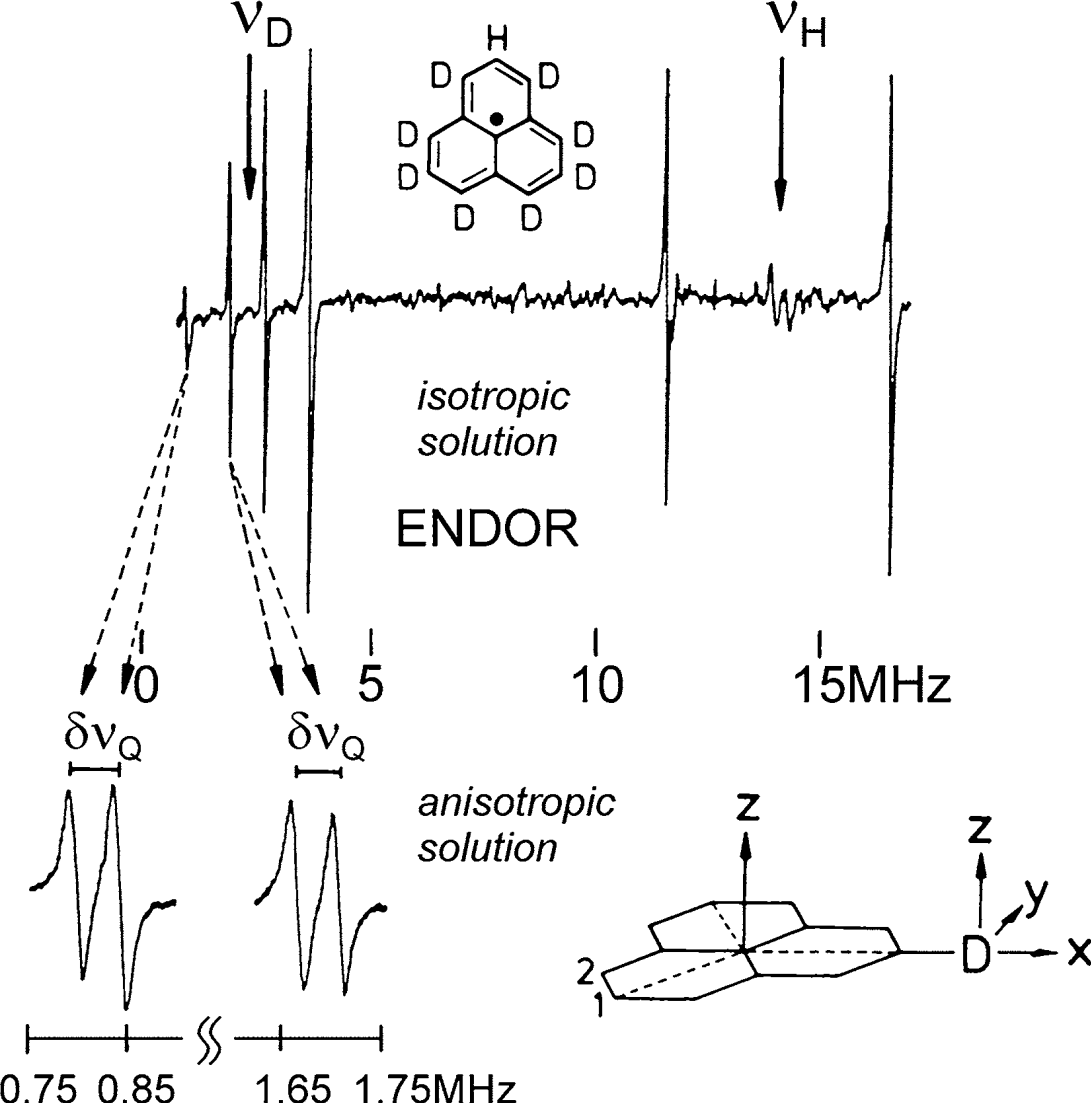
Proton and deuterium ENDOR lines of the partially deuterated perinaphthenyl radical in isotropic and anisotropic phases of a liquid crystal. In the nematic mesophase, quadrupole splittings of the deuterons, *δν*_Q_, are resolved. For both lines *δν*_Q_ = 42.2 kHz at 20 °C. From Eq. (2) with *O*_*zz*_ = − 0.300 at 20 °C, *e*^2^*q*_*zz*_*Q*/*h* = − 94 kHz (corresponding to *e*^2^*q*_CD_*Q*/*h* = + 188 kHz) was determined. Adapted from Ref. [74]

Even the small deuterium quadrupole coupling along the C–D bond of the partially deuterated and perdeuterated aromatic radical perinaphthenyl (PNT), *e*^2^*q*_CD_*Q*/*h* = + 188 kHz (error ± 3 kHz), could be measured with this technique by R. Biehl et al. [74]. The deuterated samples were kindly provided by H. Kurreck from the Organic Chemistry Institute of FU Berlin. The synthesis of deuterated PNT and additional ENDOR-in-liquid-crystal experiments of the Kurreck group are described elsewhere [75, 76]. For small quadrupole couplings of radicals in an anisotropic matrix, ENDOR is probably the only method of choice. As an illustrative example, the ^2^H ENDOR spectra of the partially deuterated PNT radical in isotropic and nematic solution [74] are presented in Fig. 11. A TM_110_ cylindrical cavity was designed to perform the electron–nuclear double- and triple-resonance experiments with high rf power.

## Selected High-Field Frozen-Solution ENDOR, EDNMR and PELDOR Experiments

### ENDOR and Orientation Selectivity in Frozen-Solution Samples

We start this section by quoting Jim Hyde who has pioneered orientation selectivity in powdered paramagnetic samples with sufficiently large Zeeman-interaction anisotropy by means of ENDOR [77]: “One day as I studied the ENDOR line shape from the four most strongly coupled protons of the tetracene positive ion under conditions of slow rotational diffusion, the idea occurred to me that ENDOR in the limit of no motion such as powders or frozen solution should be possible. The concept was to select molecules that are similarly oriented by observing a turning point with EPR and sweeping the rf to obtain single-crystal-like ENDOR spectra. And it worked! [78, 79]. Today there may be as many ENDOR experiments performed in powders as in single crystals.”

One of the most important advances in EPR and ENDOR spectroscopy has been the extension from X-band EPR to high magnetic fields and microwave frequencies, for example W-band EPR, very much in analogy to what happens in NMR (for overviews, see e.g., [2, 8, 80–84]. For low-symmetry systems, particularly in frozen solution samples, standard EPR suffers from low spectral resolution due to strong inhomogeneous line broadening. Such problems arise, for instance, because several radical species or different magnetic sites of rather similar *g* values are present or the small *g*-tensor anisotropy of the paramagnetic system does not allow canonical orientations of the powder EPR spectrum to be observed. For improving the spectral resolution by high-field EPR, we have to define a lower limit of the microwave frequency and the corresponding magnetic field B_0_. For “true” high-field EPR experiments, properties of the spectrometer have to be related with properties of the sample: For all cases of delocalized spin systems, in which unresolved hyperfine interactions dominate the inhomogeneous EPR linewidth, a true high-field experiment must fulfill the condition:

3 $${\frac{\Delta g}{{ \, g_{\text{iso}} }} \cdot B_{ 0} > \Delta B},$$

i.e., the anisotropic electron Zeeman interaction, described by the difference ∆*g* of principal *g*-tensor components, must exceed the inhomogeneous EPR linewidth ∆*B*. On the other hand, when ∆*B* is reduced by isotope labeling, e.g., by perdeuteration of the nitroxide spin-label, lower *B*_0_ fields may already be sufficient to meet the condition for true high-field EPR of Eq. (3).

**Fig. 12:**
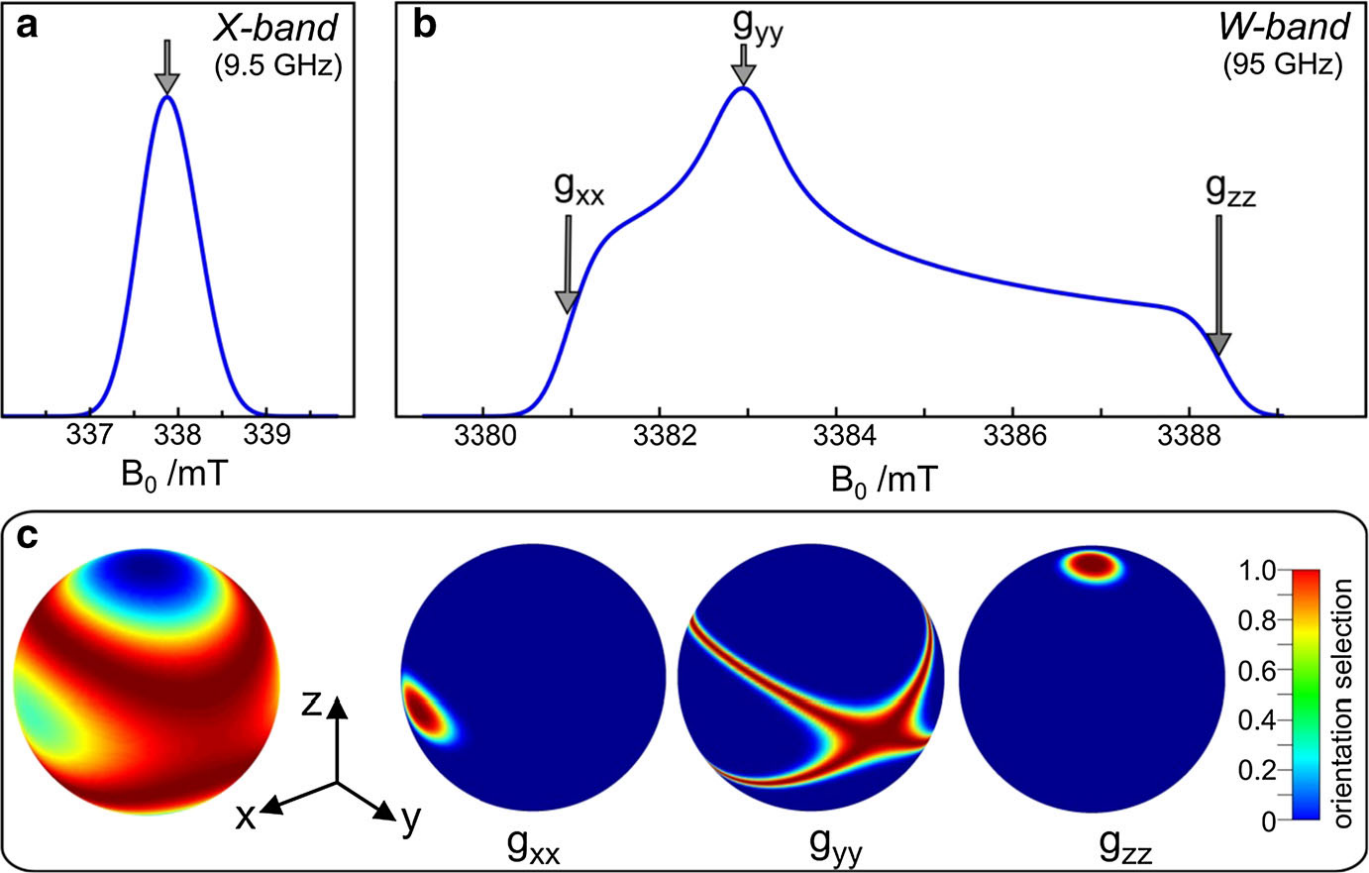
Enhanced orientation selectivity by high-field EPR, taking the anion radical of the ubiquinone acceptor cofactor in frozen-solution bacterial photosynthetic reaction centers as example. The important feature of enhanced orientation selectivity in high-field EPR for randomly oriented spin systems becomes essential for organic radicals with small *g*-anisotropies. Well below room temperature, the overall rotation of a protein complex, for example, becomes so slow that powder-type EPR spectra are obtained. If the anisotropy of the leading interaction in the spin Hamiltonian is larger than the inhomogeneous linewidth, the canonical orientations of the dominating interaction tensor can be determined even from disordered powder-type samples. As a consequence, single-crystal like information about the hyperfine interactions can be extracted by performing orientation-selective ENDOR or PELDOR at the magnetic field values corresponding to resolved spectral features. **a** X-band EPR without orientation selection of canonical *g*-tensor components; **b** W-band EPR with partial orientation selection of the canonical *g*_*xx*_, *g*_*yy*_, *g*_*zz*_ components of the *g*-tensor; **c** calculated orientation selections mapped on the unit sphere

Another shining example is the enhanced orientation selectivity of high-field EPR in disordered samples, as illustrated in Fig. 12. This feature becomes essential for randomly oriented spin systems with a small *g*-anisotropy and without transition-metal hyperfine anisotropy, like organic radicals in frozen solutions or biological cofactors in photosynthetic reaction center proteins. Far below room temperature, the overall rotation of a protein complex is so slow that powder-type EPR spectra are obtained. If the anisotropy of the leading interaction in the spin Hamiltonian is larger than the inhomogeneous linewidth, the canonical orientations of the dominating interaction tensor can be determined, even from disordered powder-like EPR spectra with Pake patterns that are familiar to NMR spectroscopists.

As a consequence of the magneto-selections of the tensor orientations, by means of a double-resonance experiment single-crystal like information about hyperfine interactions can be extracted by performing orientation-selective ENDOR at the field values of resolved spectral features of the powder pattern. Similar arguments hold for dipolar electron–electron spin interactions in radical pairs when studied by orientation-resolving high-field PELDOR (see below). As was shown by Jim Hyde and co-workers in the years 1968–1970 [79, 85, 86], in the case of transition-metal complexes the large hyperfine anisotropy of the metal ion may provide this orientation selectivity for the entire orientation distribution of the molecules. Often, their *g*-anisotropy is large enough to allow for distinct orientation selectivity even in standard X-band EPR (in a Zeeman field of only 0.3 T) allowing one to obtain single-crystal-like ENDOR [79, 85, 86]. The best approach for elucidating molecular structures and orientation in detail is, of course, to study single-crystal samples. Unfortunately, to prepare them for large biological complexes such as membrane proteins is often difficult or even impossible.

### Conformational Changes During Light-Induced Electron Transfer in Photosynthetic RCs?

The flexibility of the quinone binding site Q_A_ of the bacterial photosynthetic reaction center (RC) of *Rb. sphaeroides* (see Fig. 8) has initiated speculations about its functional role in the charge-separation and charge-recombination cycle. Such speculations on potential structural changes associated with Q_A_ reduction were fostered by an early observation by D. Kleinfeld in the Feher group [87]: he showed, by optical spectroscopy, that the rate of recombination from the light-induced transient radical-pair state ${\text{P}}_{865}^{ \cdot + } {\text{Q}}_{A}^{ \cdot - }$ to the ground-state P_865_Q_A_ is different in RCs cooled to cryogenic temperatures in the dark (“dark-adapted RCs”) from those in RCs cooled under continuous illumination (“light-adapted RCs”) [87]. The authors rationalized the slower recombination kinetics in the light-adapted sample by tentatively suggesting small changes of the donor–acceptor average distance (by about 1 Å) and its distribution around the average value.

**Fig. 13:**
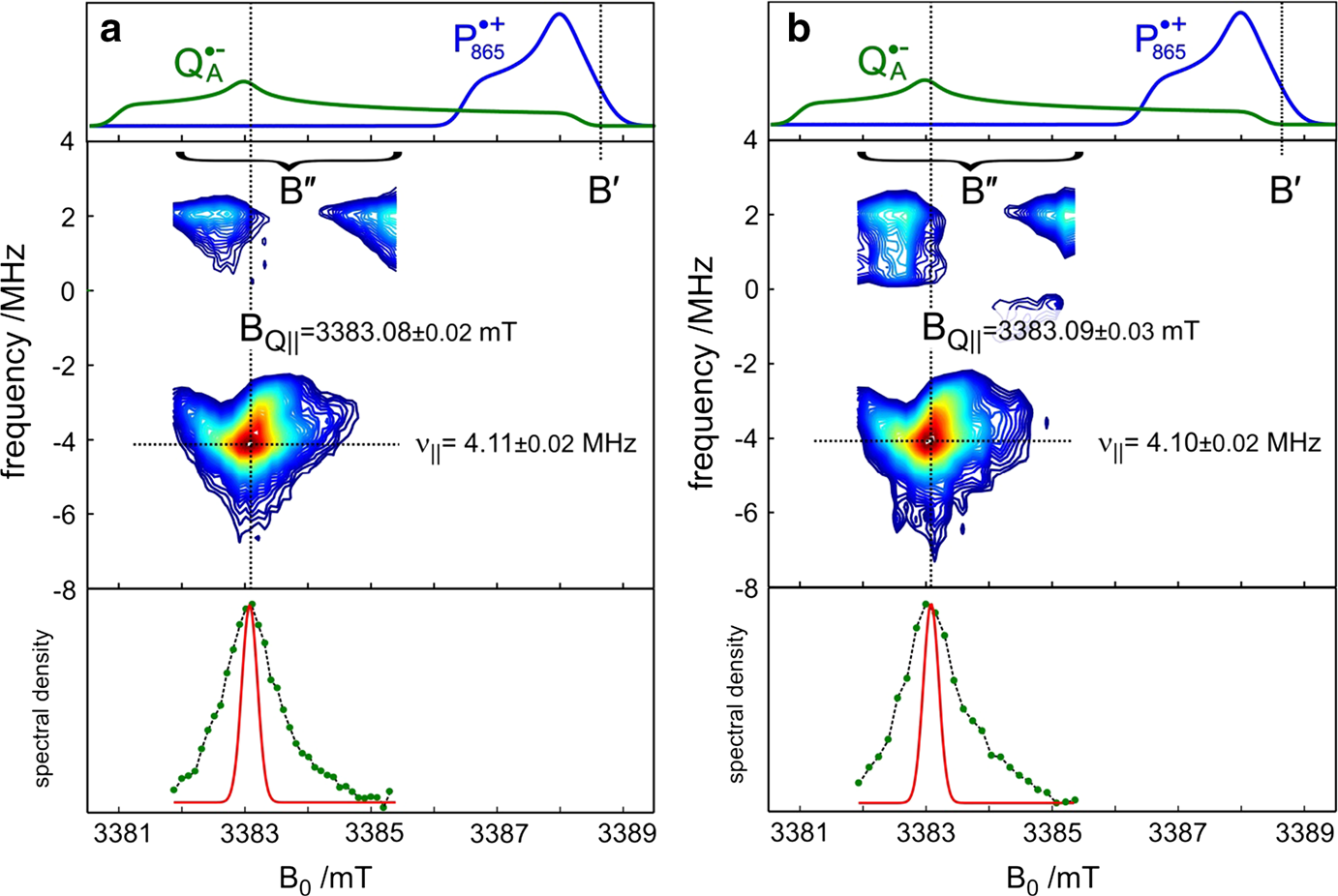
W-band PELDOR spectra of the spin-correlated radical pair ${\text{P}}_{865}^{ \cdot + } {\text{Q}}_{A}^{ \cdot - }$ in Zn-RCs (non-heme Fe^2+^ iron replaced by Zn^2+^) at *T* = 90 K, **a** in the RC sample frozen in the dark, **b** in the RC sample frozen under continuous illumination. Only those PELDOR responses are shown that were used to probe the spectral position, B_Q||_, within the ${\text{Q}}_{A}^{ \cdot - }$ EPR spectrum corresponding to the parallel dipolar frequency, *ν*_*║*_. The observer mw frequency is fixed at the value corresponding to the field value *B*′, while the pump mw is swept through the field region *B*″. Upper part: the individual EPR spectra of ${{\text{P}}_{ 8 6 5}^{ \cdot + } }$ and ${\text{Q}}_{A}^{ \cdot - }$ are shown to indicate the spectral positions at which PELDOR measurements are performed. Middle part: contour plots of the positive Fourier amplitudes of the PELDOR echo decays. Lower part: contour-plot amplitude (at the slice position) vs. magnetic field (dots). The intrinsic inhomogeneous EPR line width (red line) is 0.29 mT (note that the RC is fully deuterated allowing for such a narrow linewidth). The observed broadening of the PELDOR lines is due to an orientation distribution of ${\text{Q}}_{A}^{ \cdot - }$. For further explanations of the W-band PELDOR experiment, see [92, 94] (color figure online)

This suggestion was questioned on the basis of FTIR spectroscopy [88], X- and Q-band transient cw EPR and out-of-phase electron-spin echo (ESE) experiments [89, 90]. No significant changes in the donor–acceptor distance and its distribution were observed. On the other hand, it was proposed from an analysis of quantum-beat oscillations of transient Q-band EPR signals (*T* = 70 K) of ${\text{P}}_{865}^{ \cdot + } {\text{Q}}_{A}^{ \cdot - }$ radical pairs in dark-adapted RCs [91], that an unprecedented reorientation of Q_A_ by as much as 60° upon light-induced charge separation would occur. The authors concluded that this large difference in orientations reflects a rotation of the quinone in its ring plane that is caused by structure accommodation to the charged configuration of the acceptor binding site. At variance with this model [91], A. Savitsky et al. [92] and M. Flores et al. [93] concluded from their orientation-resolving W-band high-field dipolar EPR studies (PELDOR at *T* = 90 K) of the transient ${\text{P}}_{865}^{ \cdot + } {\text{Q}}_{A}^{ \cdot - }$ in dark-adapted RCs that no significant rearrangement at the Q_A_ site occurs under illumination; see Fig. 13. We make the point that the apparent discrepancy of these studies is rooted in the inherent sign ambiguity of the magnetic-resonance measured squares of any spin-interaction tensors and the resulting degeneracy of structure solutions that has to be properly considered [92].

In Fig. 13, the PELDOR results for the dark-adapted and light-adapted RCs are summarized. The parameters *ν*_*║*_ and *B*_*Q*║_, which are highly specific for the radical-pair structure (*ν*_*║*_ for the distance, *B*_*Q*║_ for the orientation, see [92]), can be directly read off from the PELDOR spectra. The values *ν*_*║*_ = 4.11 ± 0.02 MHz and *B*_*Q*║_ = 3383.08 ± 0.02 mT for the dark-adapted sample fully agree with *ν*_*║*_ = 4.10 ± 0.02 MHz and *B*_*Q*║_ = 3383.09 ± 0.03 mT for the light-adapted sample. Also the angular distribution width, Δ*B*_1/2_, is the same within experimental error. This implies that neither the interspin distance in the radical pair nor the relative orientation of donor and acceptor ions is different for the different illumination-freezing protocols. In other words, from PELDOR we learn that there is no conformational redistribution of Q_A_ under light-driven reduction.

This conclusion is fully supported by the Davies-type pulsed Q-band ^1^H ENDOR experiment: In the dark-adapted and light-adapted RCs, the ENDOR spectra of ${\text{Q}}_{\text{A}}^{ \cdot - }$ are identical within experimental error. The ENDOR results irrefutably show that independent of the history of the freezing and illumination of the RCs, ${\text{Q}}_{\text{A}}^{ \cdot - }$ remains linked to the protein by two asymmetric H-bonds from His M219 and Ala M260 to the carbonyl oxygens. This conclusion is consistent with earlier ENDOR measurements [95] and DFT calculations [96] of the H-bonding network of the quinone acceptor. This is a remarkable result as it shows that the primary quinone acceptor Q_A_ is initially located in an orientation that is already favorable for its predestined reduction by primary photosynthetic electron transfer! Such a target-oriented structural arrangement diminishes the reorganization energy for fast quinone reduction and promotes high quantum efficiency of unidirectional A-branch electron transfer.

### Site-Directed Spin-Labeled Protein Bacteriorhodopsin

Photosynthesis, the strategy for harvesting sunlight as energy source for synthesizing ATP and reducing CO_2_ to carbohydrates, has been invented by nature twice: (1) in the photosynthetic reaction-center protein complexes of certain purple bacteria and cyanobacteria, the photosynthetic process is initiated by light-induced primary electron transfer between chlorophyll and quinone cofactors, mediated by weak cofactor-protein interactions with the cellular microenvironment. (2) In the bacteriorhodopsin protein complex, the photosynthetic process is set going by light-initiated primary proton transfer between amino acid residues, mediated by conformational changes of the only cofactor, the retinal.

In this section, we review site-directed spin-label high-field EPR work on paradigmatic proton-transfer proteins such as bacteriorhodopsin (BR), the renowned “light-driven proton pump”. This nitroxide spin-label work was a cooperation project with Heinz-Jürgen Steinhoff and his group at the University of Osnabrück [97–101]. The aim of this project was to obtain new insights concerning the molecular mechanisms of light-driven proton transfer, in particular by probing site-specifically local polarity and proticity values along the proton channel in proteins embedded in functional membranes. We focus on proton-transfer intermediates of selectively MTS-labeled BR mutants from *Halobacterium* (*H.*) *salinarium* to determine potential barriers and molecular switches for vectorial transmembrane proton transfer.

**Fig. 14:**
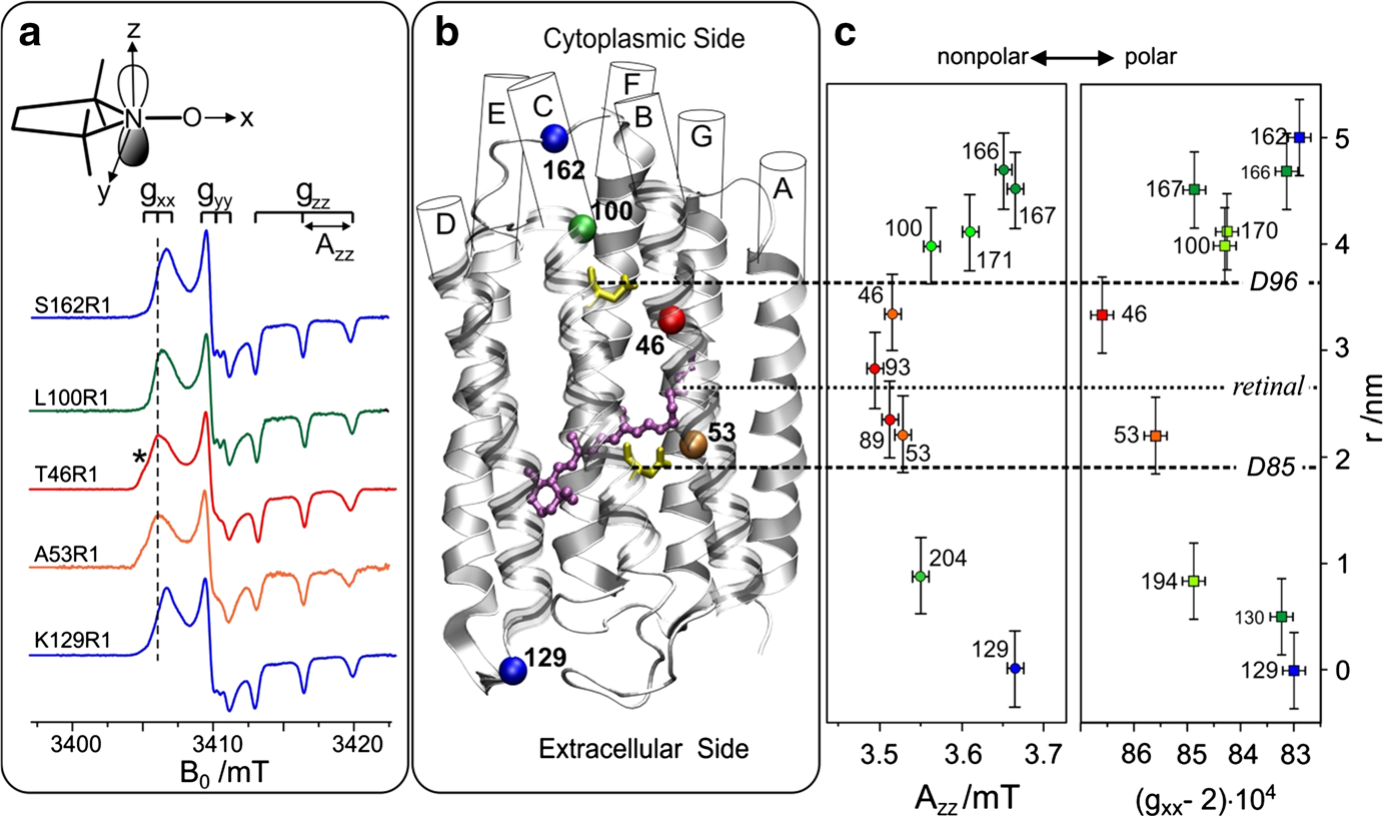
**a** Experimental W-band cw EPR spectra for a set of bacteriorhodopsin (BR) mutants with the MTS spin-labeled nitroxide side chain (R1). **b** Structural model of BR. The C_α_ atom of the spin-labeled residues, seven α-helices A to G, the chromophore retinal and D96 and D85 participating in the H^+^ transfer are indicated. **c** The magnitude of the tensor elements *A*_*zz*_ and *g*_*xx*_ of the spin labels are plotted as function of the nitroxide location in the protein with respect to position 129. For details, see [97]

By 95 GHz (W-band) high-field EPR, details of the polarity profile along the putative proton channel were probed by *g*- and hyperfine-tensor components from a series of 10 site-specifically nitroxide spin-labeled BR mutants, with MTS spin label as the reporter side chain R1 [97]. Previous studies of a large number of spin-labeled proteins have shown that the *A*_*zz*_ component of the nitrogen-hyperfine tensor and the *g*_*xx*_ component of the *g*-tensor are particularly sensitive probes of the microenvironment of the nitroxide side chain R1. They allow one to measure changes in polarity and proticity of the protein, in other words: *g*_*xx*_ and *A*_*zz*_ probe the local electric fields and the availability of H-bond forming partners of nearby amino acid residues or water molecules [97–99]. Moreover, the dynamic properties of the nitroxide side chain and, thus, the EPR spectral lineshape have been shown to contain direct information about motional constraints that are introduced by the secondary and tertiary structures of the protein in the vicinity of the nitroxide binding site [97, 98]. For measuring the polarity changes, W-band EPR spectra were recorded at temperatures below 200 K to avoid motional averaging of the anisotropic magnetic tensors. At these temperatures, R1 can be considered as immobilized on the EPR time scale. The spectra of selected mutants are shown in Fig. 14a. They exhibit the typical nitroxide powder-pattern lineshape expected for an isotropic distribution of diluted radicals. The spectra are clearly resolved into three separate regions corresponding to the components *g*_*xx*_, *g*_*yy*_ and *g*_*zz*_, the latter with resolved *A*_*zz*_ splitting. The variations of *g*_*xx*_ and *A*_*zz*_ with the nitroxide binding site can be measured with high precision.

The plots of *g*_*xx*_ and *A*_*zz*_ versus the R1 position *r* along the proton channel (Fig. 14c) demonstrate distinct variations in the polarity and proticity of the nitroxide microenvironment. According to the BR structure model [102], residue S162R1 is located in the E–F loop at the cytoplasmic surface, whereas residue K129R1 is positioned in the D–E loop on the extracellular surface. The high polarity in the environment of these residues is clear evidence that the nitroxides are accessible to water, which is in agreement with the structure. The environmental polarity of the nitroxide at positions 100, 167 and 170 is significantly smaller and reaches its minimum at position 46 between the proton donor D96 and the retinal. The plots directly reflect the hydrophobic barrier that the proton has to overcome on its way through the protein channel.

### ELDOR-Detected NMR and ENDOR on Nitroxides

**Fig. 15:**
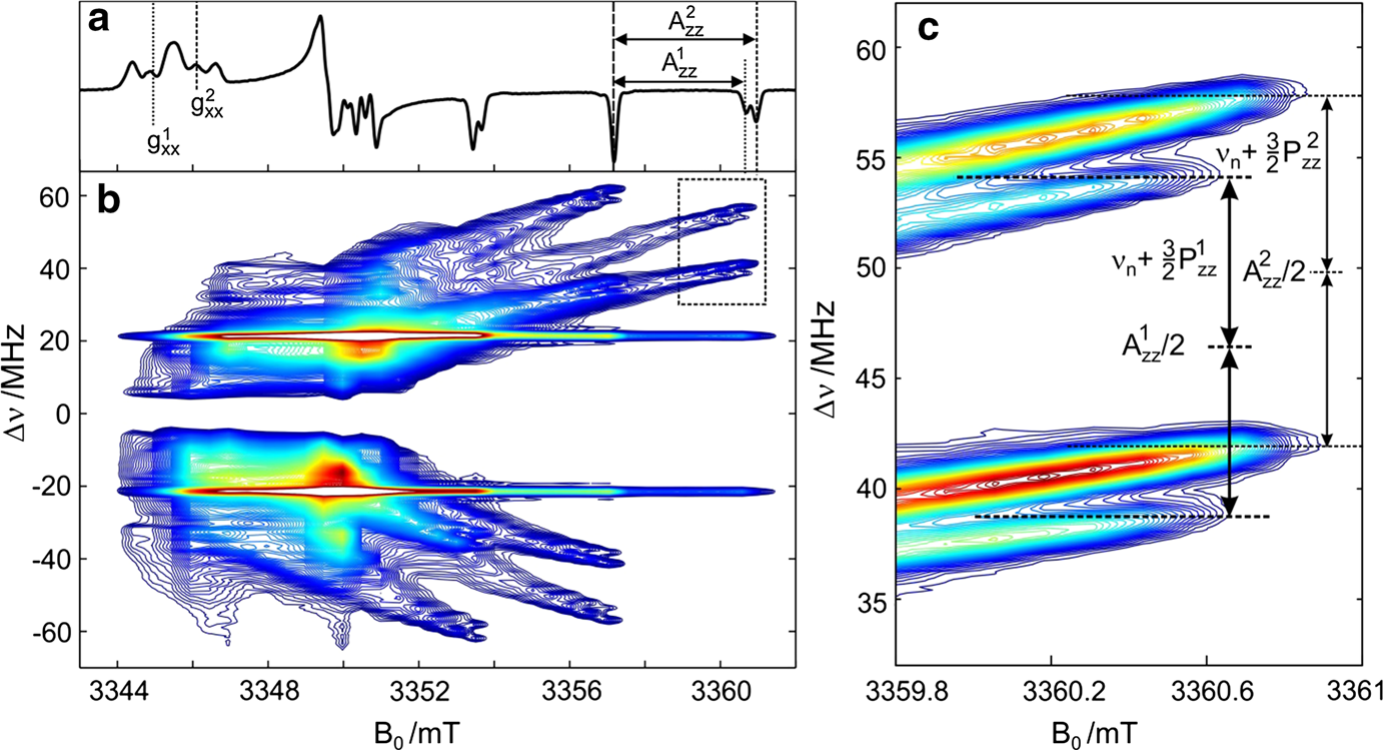
**a** Experimental W-band cw EPR spectra of 1 mM deuterated R1 in frozen solution of 2-propanol-D_8_ recorded at 90 K. The spectral positions that correspond to different principal *g*-tensor and *A*_*zz*_ values are indicated by dashed and dotted lines, respectively. **b** Contour plot of the field-frequency dependence of experimental W-band EDNMR intensities of 1 mM deuterated pyrroline-type nitroxide radical in 2-propanol-D_8_ glass at 50 K. The contour lines are shown as isohypses from 0.01 to 1 of the maximum ^14^N EDNMR intensity. **c** The EDNMR spectrum recorded at 12 magnetic field values around the *g*_*zz*_, *M*_*I*_ = − 1 spectral position, as indicated in **b**. For details, see [105]

Site-directed spin labeling using nitroxide radicals has opened up the possibility to probe the local polarity and proticity distribution in a protein with cw EPR. In frozen solution, the high-field EPR spectra reveal the *g*-tensor and nitrogen-hyperfine tensor components, which are sensitive to the microenvironment of the nitroxide spin label [97, 103, 104]. At *W*-band microwave frequency, all *g*-tensor components of the nitroxide radical become resolved. The *g*_*xx*_ region of the EPR spectra of nitroxides in frozen polar/protic solution shows, besides nitrogen hyperfine/quadrupole structure, different components related to distinct local environments. Corresponding differences of the nitrogen-hyperfine couplings *A*_*zz*_ are expected, but remain hidden in the inhomogeneously broadened EPR lines. For nitroxide-labeled proteins the *g*_*xx*_ line at W-band reveals a complex substructure indicating several spectral contributions with different *g*_*xx*_ values. For instance, the W-band cw EPR spectrum of the T46R1 bacteriorhodopsin mutant (see Fig. 14) shows a clear shoulder in addition to the main *g*_*xx*_ line as indicated the asterisk in Fig. 14a. Figure 15a (top trace) shows the W-band cw EPR spectrum of the deuterated pyrroline-type nitroxide radical (3-hydroxymethyl-2,2,5,5-tetramethylpyrrolin-1-oxyl, R1) in frozen solution of 2-propanol-D_8_. The complete perdeuteration of the system causes a considerable reduction of the EPR linewidth. Two lines are resolved at *g*_*zz*_, M_I_ = ± 1, which correspond to two nitroxide radical fractions with different *A*_*zz*_ values. The EPR spectrum was analyzed by numerical solution of the spin Hamiltonian using two parameter sets for the two nitroxide fractions. The spectral intensities of the two contributions yield the weights of the nitroxide subensembles (1) and (2) of 0.40 and 0.60, respectively. The different weights of the two radical fractions allow to determine the *g*_*xx*_ and corresponding *A*_*zz*_ values: $g_{xx}^{1}$ = 2.00911 ± 0.00005, $g_{xx}^{2}$  = 2.00843 ± 0.00005; $A_{zz}^{1}$  = 93.2 ± 0.3 MHz, $A_{zz}^{2}$  = 99.9 ± 0.3 MHz [105].

Thus, also in the case of proteins, distinct *A*_*zz*_ components are expected for each respective *g*_*xx*_ value. In cw EPR spectra they can only be resolved if the protein is perdeuterated which, however, will be an expensive endeavor. Hence, an advanced pulsed EPR method is worth striving for by which one would get around the necessity for perdeuteration and would directly probe nuclear transitions in the frequency domain. In principal, *A*_*zz*_ values can be obtained from an ENDOR experiment. In the nitroxide case, ENDOR suffers from low sensitivity and line-amplitude distortions by hyperfine enhancement effects. It turned out that ELDOR-detected NMR (EDNMR) is the method of choice. The microwave double-resonance method electron–electron double resonance (ELDOR) [106] was introduced more than 50 years ago for investigating relaxation processes in paramagnetic systems [107–112]. In continuous-wave realization this method was soon recognized, in particular by Jim Hyde [10], to be applicable also for obtaining information about the distance between paramagnetic centers. In the 1980s, ELDOR in pulsed realization (called PELDOR or DEER) was introduced [113]. Nowadays it has become a routine experiment for measurements of distances and their distributions in site-directed spin-labeled protein systems, for review see [114, 115].

In 1994, Arthur Schweiger demonstrated the applicability of pulsed ELDOR spectroscopy for the measurement of electron–nuclear hyperfine interactions and introduced ELDOR-detected NMR as an ELDOR variant of ENDOR [116]. Historically, EDNMR has suffered from the disadvantage that weakly coupled low-γ nuclei remain undetected when performing the EDNMR experiment at low microwave frequencies (X, Q-band). This problem can be avoided by performing the experiment at higher microwave frequencies, for instance at W-band. However, it was only recently that the EDNMR methodology had been used for studying real chemical systems, for review see [117–119].

The W-band EDNMR spectrum of R1-D_16_ in 2-propanol-D_8_ as function of the external magnetic field is shown in Fig. 15b [105]. In the *g*_*zz*_ spectral region, ridges are formed which end up in well-resolved lines at the spectral positions corresponding to the principal *A*_*zz*_ values of the nitroxide fractions. The analysis of the high-field EDNMR spectra yields the precise hyperfine, *A*_*zz*_, and quadrupole, *P*_*zz*_, couplings, Fig. 15c, and the corresponding weight factors of the contributing nitroxide fractions. Thus, EDNMR allows *A*_*zz*_ and *P*_*zz*_ values to be used for characterizing the nitroxide spin label microenvironment. Importantly, these parameters can be precisely determined by W-band EDNMR experiments even in protonated systems, i.e., without requiring perdeuteration [105].

**Fig. 16:**
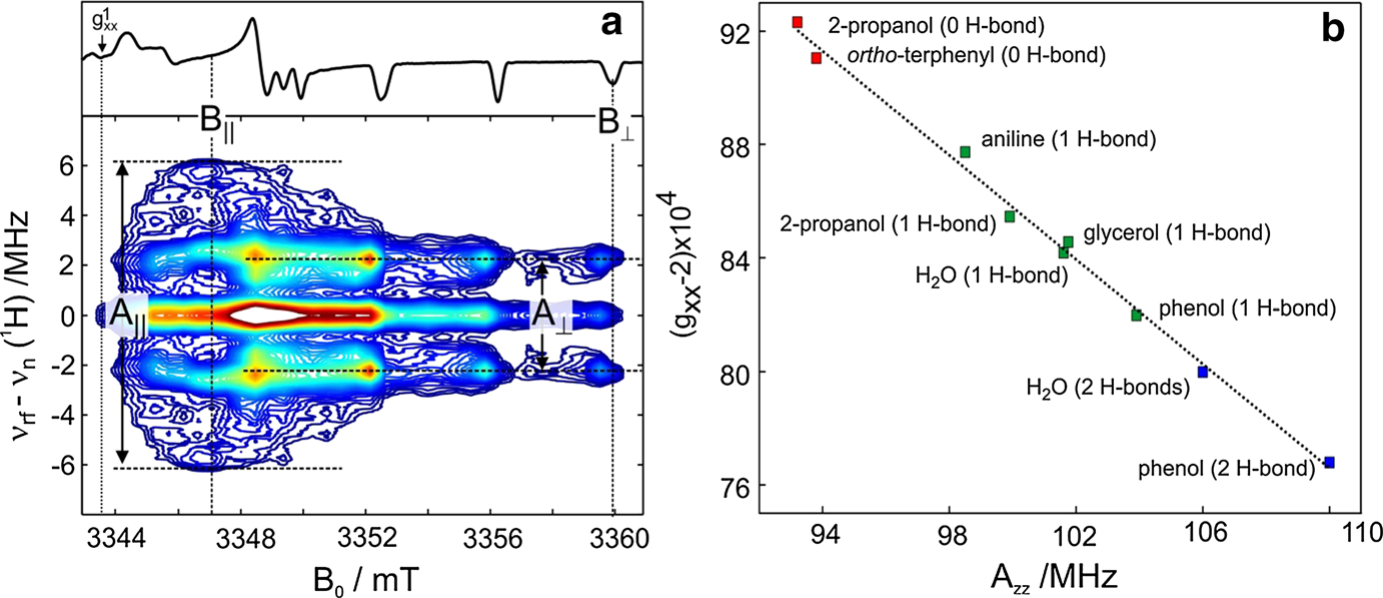
**a** The experimental W-band proton Davies-type ENDOR of 1 mM deuterated R1 in 2-propanol-D_7_(OH) glass at 60 K. The contour lines are shown as isohypses from 0.05 to 1 of the maximum ENDOR intensity due to a bonded proton. On top, the two-pulse echo-detected W-band EPR spectrum is shown in first derivative representation. **b** Plot of *g*_*xx*_ vs *A*_*zz*_ for the spin probe R1-D_16_ dissolved in various solvents. For details, see [120]

The two nitroxide fractions in frozen isopropanol with different magnetic parameters can be explained by the existence of two distinctly different nitroxide microenvironments. As the frozen isopropanol matrix represents a homogeneous glass, we suppose that the difference in microenvironments is due to formation of a hydrogen bond between the nitroxide and the proton of the OH-group of the solvent molecule. The approval of this hypothesis stems from the results of our recent ^1^H-ENDOR experiments [120]; see Fig. 16.

As is seen in the top panels of Figs. 15a and 16a, the *g*-anisotropy of the nitroxide radical is clearly resolved by W-band EPR. Thus, the resonance frequencies of hydrogen-bonded protons with predominantly dipolar hyperfine interaction are selectively recorded across the EPR spectral positions; they are related to the nitroxide molecular frame by way of the principal *g*-tensor values. The orientation-resolved ENDOR spectrum, Fig. 16a, demonstrates the characteristic dipolar hyperfine interaction pattern (‘‘butterfly’’ like). The most pronounced ridge at about ± 2.5 MHz is formed across the full EPR spectral range. It corresponds to the perpendicular hyperfine interaction frequency, *A*_⊥_. An additional ridge is formed at the field position B_*║*_ between *g*_*xx*_ and *g*_*yy*_ with the singularity at about 6 MHz corresponding to the parallel hyperfine frequency, A_*║*_. Only ENDOR responses from the remote protons [matrix line at *ν*_n_(^1^H)] are detected at $g_{xx}^{1}$. This leads to the conclusion that the second nitroxide fraction has to be ascribed to nitroxide radicals with an H-bond to 2-propanol. Moreover, the analysis of the ENDOR spectrum allows one to derive the geometrical structure of the H-bonded bimolecular system which represents a σ-type complex in which the hydrogen bond is formed with the participation of the lone-pair electrons on the oxygen atom of the nitroxide radical [120].

Figure 16b correlates *g*_*xx*_ and *A*_*zz*_ values obtained for the R1 nitroxide in a variety of organic solvents by using 244 GHz cw EPR and W-band EPR methods as well as W-band ENDOR to support the identification of hydrogen-bonded complexes. The figure shows that the increase of *g*_*xx*_ and decrease of *A*_*zz*_ values follow the availability of proton donors of the solvent, thereby modeling the proticity of the microenvironment at the spin-labeled protein site. This new insight offers a rationale for the linear variation of averaged $\langle g_{xx} \rangle$ with $\langle A_{xx} \rangle$ that is commonly derived from cw EPR spectra [103, 104]. The slope of this averaged-value variation is significantly larger than the slope of $g_{xx}^{i}$ versus $A_{zz}^{i}$ for the individual tensor components. The small variation of each $g_{xx}^{i}$ reflects the small change of the *g*_*xx*_ value of the spin label with a definite number of hydrogen bonds in a specific protein site when changing from one protein site to another. This is in contrast to using the averaged tensor components as probes for the microenvironment because the large variation of $\langle A_{xx} \rangle$ mostly reflects the variation of matrix proticity from one protein site to the other.

## Concluding Remarks

During the last two decades, the magnetic-resonance community was a witness of a boost of new and exciting applications of multi-resonance and multi-frequency EPR spectroscopy in chemistry, biology and physics. This is largely due to technological breakthroughs in the development of pulsed microwave sources, detectors and circuit components for frequencies up to several hundred GHz, sweepable cryomagnet designs and ultra-fast data-acquisition and -handling instrumentation. This enables the EPR spectroscopists to introduce multiple-pulse microwave and radiofrequency irradiation schemes at very high Zeeman fields, very much in analogy to what is common practice now in modern NMR spectroscopy. Moreover, the combination of EPR and NMR methodologies provides novel analytical tools. They are distinguished by their unique potential for an elucidation of structure and dynamics of transient complex systems embedded in disordered matrices, e.g., electron- or proton-transfer intermediates in membrane proteins. A key role in the amalgamation of EPR with NMR was played early on by George Feher with creating solid-state ENDOR, followed by Jim Hyde, Gus Maki and Jack Freed with realizing liquid-state ENDOR and ELDOR. A little later, the catching up race of EPR in relation to NMR was raised to an even higher level of virtuosity by Arthur Schweiger with his creations of powerful combinations of pulsed microwave and radiofrequency fields [121]. He acted like a hinge between the two magnetic-resonance siblings, EPR and NMR, for example with his ELDOR-detected NMR (EDNMR) strategy. ELDOR concepts were employed also in the development of electron–electron dipolar spectroscopy (PELDOR or DEER) by Kev Salikhov, Yuri Tsvetkov and Jack Freed together with EPR colleagues involved in distance measurements from around the world [115]. Like many other inventions in pulsed EPR, also PELDOR/DEER originates from NMR analogs, in fact from a concept in solid-state NMR back in much earlier times [122].

There are at least five important features that are emerging from both EPR and NMR spectra with increasing Zeeman field: (1) enhanced spectral resolution; (2) enhanced orientation selectivity in disordered samples; (3) enhanced low-temperature spin polarization; (4) enhanced detection sensitivity for restricted-volume samples, and (5) enhanced ‘‘snapshot’’ sensitivity for probing fast motional dynamics. For example, the strategy for spectral resolution enhancement is similar in EPR and NMR: with increasing external Zeeman fields the field-dependent spin interactions in the spin Hamiltonian are separated from the field-independent ones. In high-field EPR, the *g*-factor resolution is increased in relation to the hyperfine couplings; in high-field NMR the chemical-shift resolution is increased in relation to the spin–spin couplings.

ENDOR, PELDOR or EDNMR at high Zeeman fields and microwave frequencies take additional advantage of the orientation selection of molecular subensembles in powder-type or frozen-solution samples. Thereby, even in the case of small *g-*anisotropies, these double-resonance techniques can provide single-crystal like information about electron dipolar and electron–nuclear hyperfine interactions, including the directions of inter-spin dipolar axes in coupled radical pairs or cofactor-hydrogen bonds in the protein complexes.

High-field EPR noticeably extends the applicability of the site-directed spin-labeling technique, which was originally developed for X-band EPR. Owing to the spectral resolution of both the *g*-tensor and hyperfine-tensor components of nitroxide spin probes at high Zeeman fields, polarity and proticity profiles of the protein microenvironment can be identified, for example in transmembrane channel proteins.

Both magnetic-resonance cultures, EPR and NMR, are driven by the same motivation—to understand the spin interactions in complex systems for revealing their structure and dynamics, be it from the viewpoint of material sciences, structural biology or medical sciences. The big issues in current natural and life sciences—*Health and Disease*, *Environment*, *Sustainable Energy* and *Learning from Nature*—ask for the best of all analytical methodologies to apply, these issues are valid also in the magnetic-resonance community. As Jim Hyde at the Medical College of Wisconsin has shown in an exemplary way during four decades of research and development, such a demanding task is going to be manageable only by intense collaboration between dedicated scientists from many different fields of education and expertise. MRI (magnetic-resonance imaging), Jim Hyde’s second main research and development program since the mid-1980s, led to the development of surface coils and gradient coils that are being used even in functional MRI. This is no longer exclusively a domain of the NMR people. EPR imaging is already on the way to become a powerful diagnostic tool in new-materials and medical-diagnosis laboratories. Successful contributions to such big issues will rely on the design of new magnetic-resonance experiments “off the beaten track”. We are sure that Jim Hyde will agree to this assessment.
